# A prometaphase mechanism of securin destruction is essential for meiotic progression in mouse oocytes

**DOI:** 10.1038/s41467-021-24554-2

**Published:** 2021-07-14

**Authors:** Christopher Thomas, Benjamin Wetherall, Mark D. Levasseur, Rebecca J. Harris, Scott T. Kerridge, Jonathan M. G. Higgins, Owen R. Davies, Suzanne Madgwick

**Affiliations:** 1 Biosciences Institute, Faculty of Medical Sciences, Newcastle University, Newcastle upon Tyne, UK; 2 Institute of Cell Biology, University of Edinburgh, Michael Swann Building, Max Born Crescent, Edinburgh, UK; 3 Present Address: Max Planck Institute for Biophysical Chemistry, Gottingen, Germany

**Keywords:** Chromosome segregation, Meiosis, Chromosomes

## Abstract

Successful cell division relies on the timely removal of key cell cycle proteins such as securin. Securin inhibits separase, which cleaves the cohesin rings holding chromosomes together. Securin must be depleted before anaphase to ensure chromosome segregation occurs with anaphase. Here we find that in meiosis I, mouse oocytes contain an excess of securin over separase. We reveal a mechanism that promotes excess securin destruction in prometaphase I. Importantly, this mechanism relies on two phenylalanine residues within the separase-interacting segment (SIS) of securin that are only exposed when securin is not bound to separase. We suggest that these residues facilitate the removal of non-separase-bound securin ahead of metaphase, as inhibiting this period of destruction by mutating both residues causes the majority of oocytes to arrest in meiosis I. We further propose that cellular securin levels exceed the amount an oocyte is capable of removing in metaphase alone, such that the prometaphase destruction mechanism identified here is essential for correct meiotic progression in mouse oocytes.

## Abstract (web summary)

Securin inhibits the protease separase and must be removed before anaphase to ensure timely chromosome segregation. Here, the authors define a mechanism of securin destruction in prometaphase I in mouse oocytes and demonstrate its importance for successful meiotic progression.

## Introduction

Successful cell division depends on the ordered degradation of key cell cycle proteins. In both mitosis and meiosis, this degradation relies on the activity of the anaphase-promoting complex (APC/C)^1^. The APC/C recognises its substrates through short linear motifs known as ‘degrons’^2^. Once recruited, APC/C substrates are ubiquitinated and delivered to the 26S proteasome for degradation. The ordering of APC/C substrate recruitment is determined by the degrons present within a substrate, as well as by the composition and post-translational modification of APC/C subunits. A principal factor determining which substrates are targeted at a given time is the identity of the co-activator protein (Cdc20 or Cdh1) present at the APC/C^3^.

Securin is an APC/C substrate in both mitosis and meiosis^4,5^. Prior to its degradation, securin functions to inhibit the protease separase which, when activated, cleaves the cohesin rings that hold chromosomes together^6–17^. The inhibitory function of securin is mediated through direct binding of its C-terminal region or ‘SIS’ (separase-interacting segment) across the surface of separase^18–20^. The SIS is known to contain a pseudosubstrate motif that sits within the active site of separase. Indeed, when residues within the pseudosubstrate motif in yeast securins are switched to match equivalent residues in the separase substrate Scc1, securin is efficiently cleaved^9,21^. In addition, securin contains a conserved LPE (leucine-proline-glutamic acid) motif upstream from the pseudosubstrate motif but also within the SIS. An LPE motif is also found in Scc1 and was shown to enhance cleavage by separase through a docking interaction away from the active site. However, securin’s LPE motif blocks this interaction, providing a second mechanism by which securin-separase binding directly inhibits cohesin cleavage^22^.

Alongside a critical role in driving chromosome alignment, cyclin B1-Cdk1 activity has also been shown to inhibit separase. This inhibition is mediated by Cdk1 phosphorylation of separase and subsequent complex formation between cyclin B1 and separase^23–26^. The binding of separase with either securin or cyclin B1 is mutually exclusive, and the relative contribution of each inhibitory pathway varies depending on cell type and developmental state^24,27^. In fixed meiosis I (MI) mouse oocytes, it has been reported that either securin or cyclin B1-Cdk1 mediated separase inhibition may be perturbed independently without an increase in premature chromosome separation^28^. Errors in segregation were only detected when both inhibitory pathways were removed. Interestingly, however, the situation is different in meiosis II where securin depletion alone leads to complete chromatid separation, suggesting a change in the way separase is inhibited between the two meiotic cycles^28,29^. Importantly though, while securin may be dispensable in some cells, it must be removed ahead of anaphase to ensure the timely release of separase activity^4,30^. Furthermore, the degradation of securin must be coupled to the degradation of cyclin B1^30–32^. This is to ensure that separase activation and the poleward movement of chromosomes, triggered by a loss of cyclin B1-Cdk1 activity, are synchronous.

In mitosis, the synchronous loss of securin and cyclin B1 is ensured by the similarity of their destruction mechanisms. Both are ubiquitinated by the APC/C during metaphase^4,33^. Critically, this degradation relies on both the availability of the APC/C co-activator protein Cdc20 and on the D-box motif present within the N-terminus of both securin and cyclin B1^34–36^. Once all chromosomes are properly attached to spindle microtubules in metaphase, Cdc20 and the APC/C form a bipartite D-box receptor^37^. Prior to this, Cdc20 is sequestered by the spindle assembly checkpoint (SAC); a diffusible signal generated at each unattached kinetochore. The SAC thus functions to block the formation of the APC/C-Cdc20 bipartite D-box receptor, preventing securin and cyclin B1 destruction until all chromosomes are attached to spindle microtubules^38–40^. In contrast, where APC/C substrates must be targeted prior to metaphase alignment, additional motifs are present that function to bypass the SAC. One example of this is the ABBA motif in cyclin A, which directly outcompetes SAC protein binding of Cdc20^41,42^. Through this mechanism, the ABBA motif in cyclin A permits D-box-dependent degradation in prometaphase during an active SAC.

In contrast to mitosis, securin and cyclin B1 destruction initiate during prometaphase in mouse oocyte meiosis I, prior to full chromosome alignment^43,44^. At first, this might seem like a failure to ensure accurate chromosome segregation. However, our recent work demonstrates that cyclin B1 is present in excess of Cdk1 in prometaphase I in mouse oocytes^44^. At this time, destruction represents only the loss of non-Cdk1-bound cyclin B1. Cdk1-bound cyclin B1 is preserved, prolonging the period over which high Cdk1 activity is present in the oocyte. The degradation of non-Cdk1-bound cyclin B1 in prometaphase I thereby represents a key feature of a mechanism that functions to prevent aneuploidy in mouse oocytes. Importantly, free cyclin B1 is targeted to the APC/C by a motif in addition to the D-box (the PM motif), which is only accessible in the unbound pool of cyclin B1. By this strategy, an excess of cyclin B1 acts as an APC/C decoy to maintain Cdk1 activity and prolong prometaphase in oocyte meiosis I. This is critical in mouse oocytes since, in the absence of excess cyclin B1, SAC activity is not sufficient to prevent anaphase for long enough to fully align chromosomes; a process that takes hours in oocyte meiosis I rather than the tens of minutes to complete mitosis^44^.

Given that securin and cyclin B1 degradation occur synchronously during prometaphase I, our cyclin B1 findings raised significant questions regarding securin degradation in oocyte meiosis.

We find that securin exists in excess of separase in oocyte meiosis I. We suggest that the excess of securin present in MI exceeds that which the oocyte is capable of removing efficiently in metaphase alone. We identify key residues within the SIS of securin that permit the removal of the non-separase-bound securin fraction in late prometaphase I. Furthermore, we present evidence that non-separase-bound securin is targeted for degradation by a previously unidentified destruction mechanism that is essential for correct meiotic progression. Where we inhibit prometaphase destruction of the securin excess, exit from meiosis I is either delayed or blocked completely.

## Results

### Securin destruction begins 2 hours ahead of separase activation in meiosis I in mouse oocytes

**Fig. 1:**
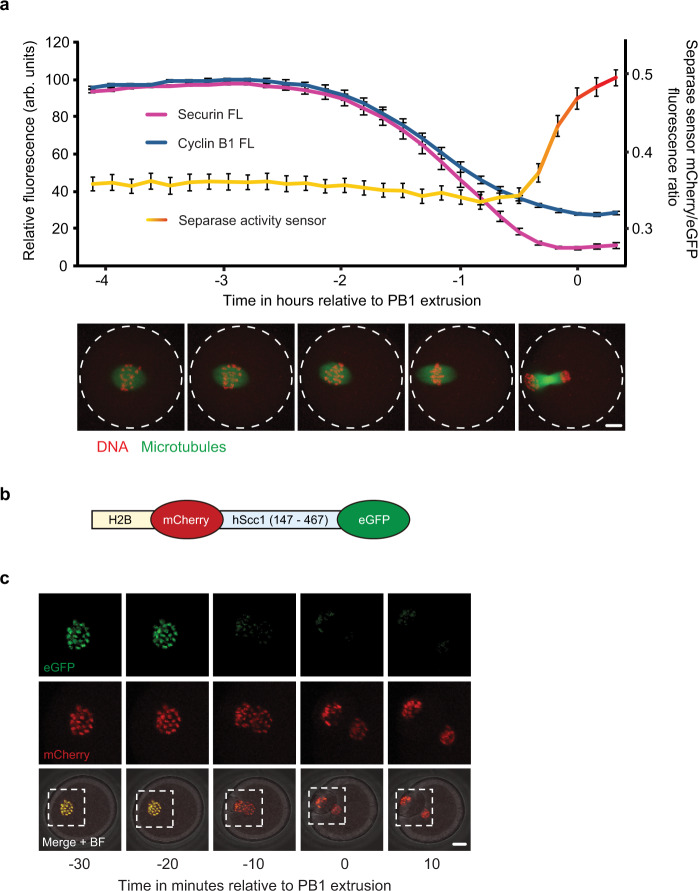
Securin destruction begins 2 hours ahead of separase activation in mouse oocyte meiosis I. **a** Graph showing the mean destruction profiles of VFP-tagged securin FL (magenta trace, *n* = 25) and cyclin B1 FL (blue trace, *n* = 62) alongside separase activity as determined by a separase activity biosensor (mCherry/eGFP ratio, *n* = 21) in MI mouse oocytes relative to PB1 extrusion. Error bars ± SEM. Representative confocal images show a maturing oocyte expressing Map4-GFP (microtubules in green) and incubated with SiR-DNA (DNA in red) at times relative to the *x* axis of the graph. Scale bar = 10 µm. **b** Schematic diagram of an H2B-mCherry-Scc1-eGFP separase activity biosensor. **c** Representative time-lapse images of an oocyte expressing the separase activity biosensor in Fig. 1b, imaged every 10 minutes at the times indicated relative to PB1 extrusion. eGFP (green), mCherry (red) and merged fluorescence + bright-field (BF) images are shown. Scale bar = 10 µm. eGFP and mCherry images correspond to the areas marked by the white dotted lines in the merge + BF images. Images displayed in **a** and **c** are representative of between 10 and 20 oocytes from experiments repeated at least three times.

In mitosis, securin is destroyed alongside cyclin B1 and only in metaphase, once the spindle checkpoint is inactivated in response to correct attachment of all kinetochores to microtubules^4,33^. In contrast, while securin and cyclin B1 are also targeted simultaneously in meiosis I in mouse oocytes, their destruction initiates much earlier, ahead of full chromosome alignment and spindle migration in prometaphase (Fig. 1a). However, as division errors are rare in mouse oocytes, it seemed unlikely that this early securin and cyclin B1 destruction affects separase activity so far ahead of anaphase. To test this, we used a separase activity biosensor generated by Nam et al. that has previously been validated in HeLa cells, mouse embryonic fibroblasts (MEFs) and oocytes^45–48^. The sensor consists of a nucleosome-targeted H2B protein fused to eGFP and mCherry fluorophores. Between the two fluorophores, an Scc1 peptide sequence is cleaved by active separase (Fig. 1b). Scc1 cleavage results in a yellow to red colour shift as the eGFP signal dissociates into the cytoplasm and mCherry remains bound to histones associated with chromosomal DNA. We injected germinal vesicle (GV) stage oocytes with mRNA encoding this biosensor and imaged cells through MI maturation. Here, we consistently observed a clear shift in colour just 20–30 minutes ahead of the first polar body (PB1) extrusion (Fig. 1c). This timing was confirmed by the quantification of the mCherry/eGFP fluorescence ratio (Fig. 1a). The data indicate that separase is only active from 30 minutes ahead of PB1 extrusion, with the majority of substrate cleavage taking place in the final 20 minutes. Importantly, this demonstrates that separase becomes active >2 hours after the initiation of securin destruction.

### The D-box is insufficient for wild-type securin destruction in MI

**Fig. 2:**
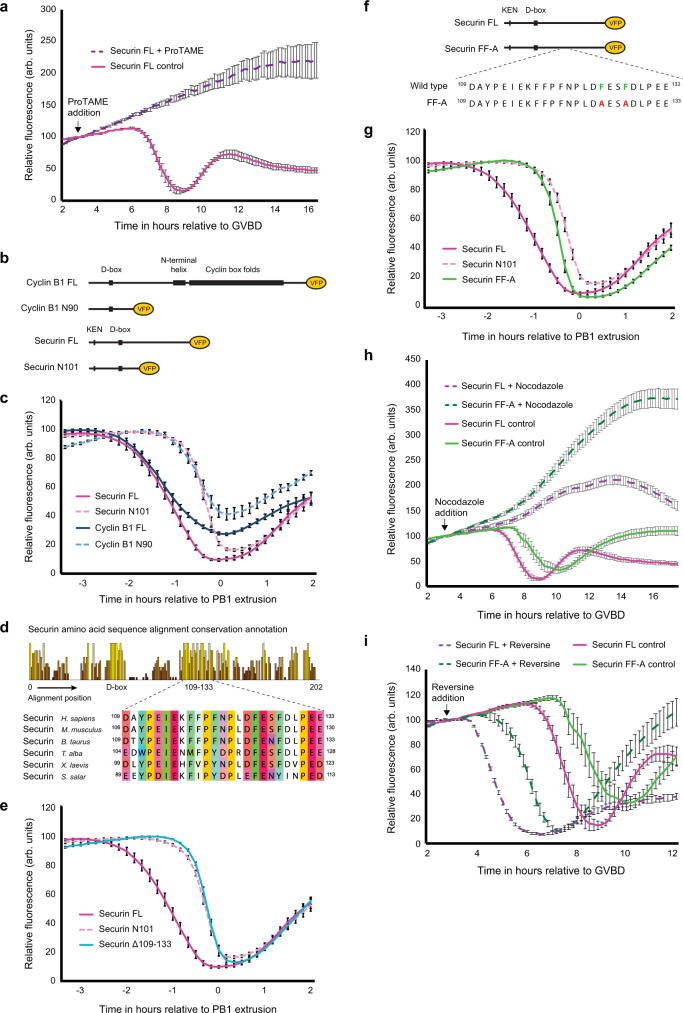
A discrete region within the C-terminus of securin promotes destruction in prometaphase. **a** Mean VFP-tagged securin FL (purple dashed trace, *n* = 11) destruction profile following incubation in 1.5 µM ProTAME to inhibit APC/C activity. Mean VFP-tagged securin FL (magenta trace, *n* = 16) destruction profile in control oocytes is included as a reference. ProTAME treated oocytes do not extrude a polar body and are therefore aligned at GVBD. **b** Schematic showing VFP-tagged securin and cyclin B1 constructs. **c** Mean securin FL (magenta trace, *n* = 25), securin N101 (pink dashed trace, *n* = 23), cyclin B1 FL (blue trace, *n* = 33) and cyclin B1 N90 (light blue dashed trace, *n* = 34) destruction profiles relative to PB1 extrusion. **d** Full sequence alignment conservation annotation and multiple sequence alignment of residues 109–133 in securin orthologs. **e** Mean VFP-tagged securin FL (magenta trace, *n* = 25), securin N101 (pink dashed trace, *n* = 23) and securin Δ109–133 (light blue trace, *n* = 23) destruction profiles relative to PB1 extrusion. **f** Schematic showing the position of Securin FF-A amino-acid substitutions. Residues F125 and F128 (shown in green in the wild-type protein) were switched to alanines (shown in red in Securin FF-A). **g** Mean VFP-tagged securin FL (magenta trace, *n* = 25), securin N101 (pink dashed trace, *n* = 23) and securin FF-A (green trace, *n* = 20) destruction traces relative to PB1 extrusion. **h** Mean VFP-tagged securin FL (magenta dashed trace, *n* = 23) and securin FF-A (dark green dashed trace, *n* = 30) destruction profiles following incubation in 150 nM nocodazole to arrest oocytes in prometaphase. Mean VFP-tagged securin FL (magenta trace, *n* = 16) and securin FF-A (green trace, *n* = 16) destruction profiles in control oocytes are included as a reference. Nocodazole treated oocytes do not extrude a polar body and are therefore aligned at GVBD. **i** Mean VFP-tagged securin FL (purple dashed trace, *n* = 16) and securin FF-A (dark green dashed trace, *n* = 16) destruction profiles following incubation in 100 nM reversine to block assembly of new SAC complexes. Mean VFP-tagged securin FL (magenta trace, *n* = 16) and securin FF-A (green trace, *n* = 16) destruction profiles in control oocytes are included as a reference. Reversine treated oocytes have an accelerated progression through meiosis and are therefore aligned at GVBD. All *n* numbers refer to the number of individual oocytes analysed over a minimum of three independent experiments. All error bars ± SEM.

In mouse oocytes, cyclin B1 is destroyed by a two-step mechanism; an initial period of prometaphase destruction requires both a D-box and an additional motif termed the PM motif. This is followed by a second period of destruction as the D-box becomes sufficient once the SAC is satisfied in metaphase^44^. Given that securin and cyclin B1 destruction is synchronous in oocytes, we reasoned that securin may also be destroyed by a similar mechanism^43,44^. We first confirmed previous findings that securin destruction in oocyte meiosis I is APC/C-dependent using ProTAME, a small molecule inhibitor shown to block APC/C activation (Fig. 2a)^5,49,50^. Then to test whether, like cyclin B1, securin destruction was biphasic, we designed and tested two fluorescent reporters; full-length securin (securin FL) and the N-terminal 101 residues (securin N101). Critically, both constructs contain the D-box and KEN box^4,30^, lysine 48^51^ and TEK boxes^52^ reported to be necessary for securin recognition by the APC/C and subsequent proteolysis (Fig. 2b). These two securin reporters, and all that follow, were coupled to Venus fluorescent protein (VFP) to give a direct readout of exogenous protein level in the oocyte.

Strikingly, we found that securin FL was consistently targeted for destruction earlier than securin N101 (~80 minutes; Fig. 2c). Furthermore, when we compared the data generated in our cyclin B1 study, we found that securin N101 destruction was restricted to metaphase, in time with a cyclin B1 N-terminus reporter (cyclin B1 N90) that contains the D-box but lacks the PM motif. This observation raised the possibility that, similar to cyclin B1 and other prometaphase APC/C substrates, an additional region is necessary to act alongside the D-box to direct wild-type securin degradation in prometaphase I in oocytes.

### A discrete region within the C-terminus of securin promotes destruction in prometaphase

To test if a second destruction motif exists within securin to facilitate prometaphase destruction, we replaced a highly conserved region located between residues 109–133 (Fig. 2d; full securin alignment is shown in supplementary fig. 1a) with a neutral 25 amino-acid TGSGP repeat linker in the full-length securin construct (securin Δ109–133). This construct was targeted for destruction in time with securin N101 (Fig. 2e) ~80 minutes after securin FL, indicating that residues between 109 and 133 are essential for a normal pattern of securin destruction in oocyte meiosis I.

Following this, we divided residues 109–133 into three groups based on sequence conservation, mutating each group to determine its importance in timing securin degradation (Supplementary fig. 1b). Based on sequence similarity with the PM motif in cyclin B1 (which centers on residues ^170^DIY^172^ in the mouse ortholog), we predicted that the first mutant (securin DAYPEIE-A) would eliminate prometaphase targeting. Surprisingly, however, securin DAYPEIE-A was instead targeted in time with securin FL (Supplementary fig. 1c). In contrast, both FFPFNP-A and DFESFD-A mutations resulted in dramatic shifts in destruction timing (Supplementary fig. 1d–e). Securin FFPFNP-A was targeted for destruction ~60 mins after securin FL but still ~20 minutes ahead of securin N101 (Supplementary fig. 1d), while securin DFESFD-A destruction mirrored that of securin N101 (Supplementary fig. 1e). This suggests that residues essential for wild-type prometaphase securin degradation lie within both of these regions.

Since securin DFESFD-A showed the most striking phenotype, we mutated a pair of highly conserved phenylalanines (F125 and F128) to alanines (securin FF-A; Fig. 2f). Here, degradation was delayed by 90 minutes, mimicking that of securin N101 (Fig. 2g; see supplementary fig. 1f for destruction timings of all mutants). Importantly, mutation of residues F125 and F128 to alanines did not impair securin binding to separase as shown by immunoprecipitation (Supplementary fig. 1g). We suggest that these two phenylalanine residues are a crucial component of a previously unidentified recognition mechanism that permits securin destruction in late prometaphase I in oocytes. In the absence of this mechanism, securin destruction initiates 90 minutes later and only in metaphase. Interestingly, these two residues and their surrounding region have no sequence similarity with the PM motif present in cyclin B1.

To confirm that the difference in degradation timing observed between securin FL and securin FF-A was not simply due to differences in protein expression, oocytes were treated with cycloheximide (CHX) to block protein synthesis (Supplementary fig. 1h). Following CHX addition, securin FL protein turnover was evident—~20% of the total protein was lost before a steep period of destruction began 4 hours later. In contrast, securin FF-A levels were relatively stable until destruction began ~5.5 hours after CHX addition. Critically, accelerated securin FF-A destruction begins ~90 minutes after securin FL, consistent with results from non-CHX-treated oocytes.

Securin FL destruction in mouse oocytes begins in prometaphase I at a time when the SAC is active as indicated by detectable Mad2 staining at kinetochores^53^. In contrast, securin FF-A destruction initiates at a later time point, once chromosomes are aligned at the metaphase plate and the spindle has migrated to the cortex (Fig. 1a). This suggests that the SAC might have less influence over securin FL than securin FF-A. To assess this, we treated oocytes with 150 nM nocodazole. This dose of nocodazole depolymerises microtubules and activates the SAC such that PB1 extrusion is blocked in >98% of oocytes. Under these conditions, securin FL degradation still occurred but was significantly reduced and delayed. In these oocytes, a gradual decline in securin FL protein levels was observed but only from ~13 hours after GVBD. In contrast, securin FF-A was largely stabilised over the same time period (Fig. 2h). Thus, while the degradation of both constructs is responsive to an increased SAC signal, securin FL is more readily turned over during an active SAC. We, therefore, questioned whether securin FL and securin FF-A would be differentially targeted when SAC signalling was removed. To assess this, we treated oocytes with 100 nM reversine to inhibit Mps1 and block assembly of new SAC complexes. As expected, reversine treatment rapidly accelerated progression through meiosis I, with securin FL degradation beginning around 30 minutes after drug addition (Fig. 2i). In the absence of SAC signalling, securin FL was still targeted for destruction significantly ahead of securin FF-A. Noticeably in both control and reversine treated oocytes, the bulk of securin FF-A degradation takes place after securin FL protein levels have been depleted (Fig. 2i).

Together our data suggest that a mechanism involving securin residues F125 and F128 functions to permit securin degradation during late prometaphase I. During this period, the D-box alone is insufficient for substrate targeting and degradation. The D-box is however still essential for both phases of securin destruction (prometaphase and metaphase) since D-box ablation perturbs destruction throughout MI and inhibits PB1 extrusion (Supplementary fig. 2b). Interestingly, when the D-box is mutated, a small amount of destruction is still observed in traces from individual oocytes. This destruction is likely mediated by the KEN box in securin, as ablation of both KEN and D-box together resulted in a more complete stabilisation (Supplementary fig. 2c). In contrast, KEN box ablation alone did not affect securin destruction (Supplementary fig. 2d).

Our data presented in Fig. 2 are consistent with a two-step securin destruction process in MI oocytes. An initial period of destruction from 3 hours ahead of PB1 extrusion requires both the D-box and an additional region, with an absolute requirement for residues F125 and F128. This is followed by the second period of destruction in metaphase as the D-box becomes sufficient for degradation 1 hour ahead of PB1 extrusion. Critically, our separase biosensor data indicate that separase is only active during the latter half of the second phase of securin destruction.

### Two phenylalanine residues key to prometaphase securin destruction are predicted to be masked when securin is bound to separase

**Fig. 3:**
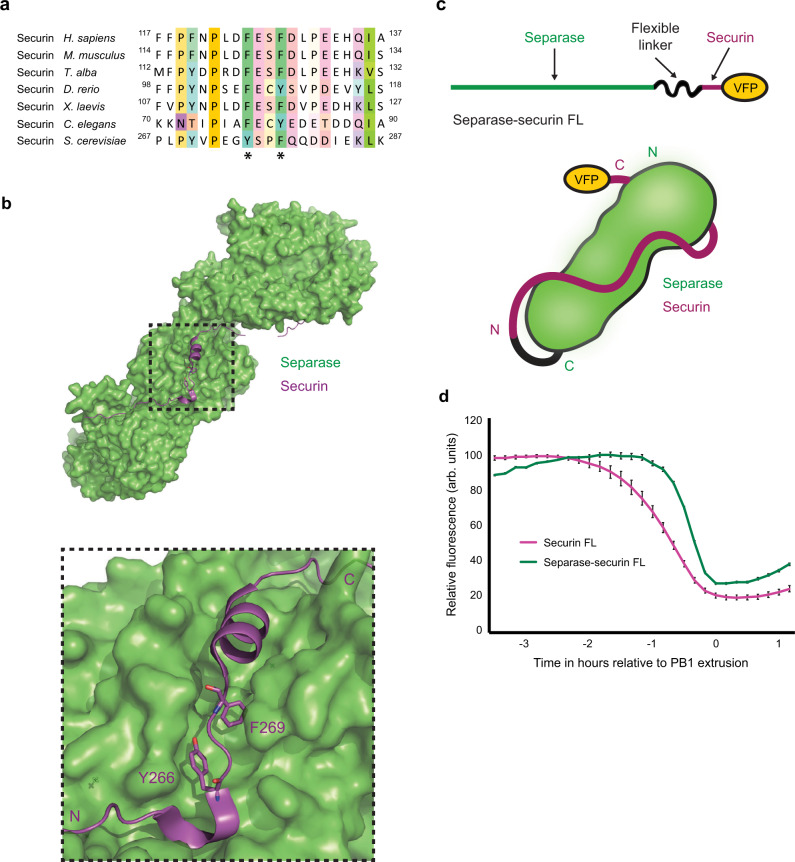
F125 and F128 are predicted to direct the prometaphase destruction of a pool of non-separase-bound excess securin. **a** Multiple sequence alignment of the region surrounding residues F125 and F128 (marked with asterisks) in securin orthologs. **b** The molecular surface of separase (green) in contact with the region of securin detailed in Fig. 3a (purple). Image generated using the crystal structure of the *Saccharomyces cerevisiae* separase-securin complex (PDB accession 5U1T [10.2210/pdb5U1T/pdb])^18^. The side chains of securin residues Y276 and F279, which correspond to F125 and F128 in the human protein, are shown as stick models and labelled in purple. The N- and C-termini of the securin segment displayed are also labelled in purple **c** Schematic representation of a tethered separase-securin complex reporter (separase-securin FL) including a cartoon demonstrating predicted complex interaction. **d** Mean VFP-tagged securin FL (magenta trace, *n* = 8) and separase-securin FL (green trace, *n* = 6) destruction profiles relative to PB1 extrusion.

Interestingly, F125 and F128 are positioned within securin’s separase interaction segment (SIS), in the vicinity of both the pseudosubstrate motif and the LPE-docking motif^18,20,22^. We therefore asked how these phenylalanine residues are positioned when securin is in complex with separase. Since a mammalian crystal structure for the securin-separase complex is yet to be solved, we instead used the structure of the *S. cerevisiae* complex as a reference^18^. Critically, these residues and the surrounding region are largely conserved between mammals and yeast (Fig. 3a). In this structure, residues Y276 and F279, corresponding to F125 and F128 in the human protein, sit deep within a hydrophobic binding pocket on the surface of separase (Fig. 3b). It is therefore highly likely that these two residues are obscured when securin is in complex with separase. The conservation of these residues throughout eukaryotes (Fig. 3a), coupled with their positioning near the pseudosubstrate region of securin, leads us to propose that both phenylalanine residues would be similarly hidden in other species.

### Securin is present in excess over separase in mouse oocytes

Given that F125 and F128 are predicted to be concealed when securin is bound to separase, we hypothesised that securin destruction in prometaphase must represent that of a non-separase-bound population.

In mitosis, securin protein is in excess of separase^31,46,54^. This ratio has been quantified in HeLa cells by immunoprecipitation experiments, where free securin is reported to be 4–5-fold more abundant than separase-associated securin^54^. We, therefore, considered that a similar excess of securin in oocytes could provide the basis for a two-step pattern of securin degradation. Given that immunoprecipitation experiments are not practical using mammalian oocyte lysates, we employed an alternative strategy to probe the securin to separase ratio. We designed and generated a construct where separase and securin are physically linked, and injected oocytes with mRNA encoding this construct (Fig. 3c). We reasoned that the resultant exogenous protein would act as a control in western blotting experiments, presenting as a larger protein than either endogenous securin or separase individually and always at a 1:1 ratio. Following extensive analysis, we propose that securin is 3–4-fold more abundant than separase in mid-prometaphase in MI mouse oocytes (Supplementary fig. 3).

In the second series of experiments using the same linked construct (separase-securin FL), we investigated how separase-binding affects the timing of securin degradation in oocytes. We reasoned that the design of the separase-securin FL construct would greatly enhance securin-separase binding relative to that between the securin-only construct (securin FL) and endogenous separase. This situation should delay the destruction of the linked securin relative to FL securin. Indeed, where we recorded the degradation profiles of separase-securin FL alongside securin FL, loss of the linked protein complex initiates ~70–80 minutes later, consistent with the timing of securin FF-A degradation (Fig. 3d).

Our data so far suggest a mechanism by which a pool of excess non-separase-bound securin is preferentially targeted for destruction in late prometaphase. This mechanism involves both the D-box and a second region within the SIS of securin where F125 and F128 are essential. Following this, a smaller pool of separase-bound securin becomes a destruction target in metaphase.

Preferential destruction of non-separase-bound securin has previously been demonstrated in HeLa cells. Hellmuth et al.^54^ showed that separase-bound securin is dephosphorylated by PP2A-B56 phosphatase, whereas free securin exists in a phosphorylated state and is a preferential APC/C target. Importantly, however, the mitotic destruction of both separase-bound and non-separase-bound securin takes place in metaphase, only after SAC satisfaction. It, therefore, seems unlikely that this same mechanism is responsible for the degradation of non-separase-bound securin in prometaphase in mouse oocytes. To assess this, we generated phospho-null and phosphomimetic securin FL and securin FF-A by mutating the four key phosphorylation sites in securin identified by Hellmuth et al. (S31, T66, S87 and S89) to either alanine (4A; phospho-null), or glutamic acid (4E; phosphomimetic). mRNA was injected encoding these constructs and destruction profiles were recorded (Supplementary fig. 4). Surprisingly, for both securin FL and securin FF-A proteins, the phosphomimetic mutation showed a delayed degradation, the opposite of its effect in mitosis. In contrast, the phospho-null mutation brought securin FF-A degradation slightly earlier but had no effect on securin FL (Supplementary fig. 4).

We therefore suggest that while phosphorylation may fine-tune the degradation timing of both constructs, the mechanism of non-separase-bound securin degradation described in this manuscript does not absolutely depend on phosphorylation status. The preferential degradation of non-separase-bound securin is more likely mediated by a steric interaction with the APC/C or its co-activator.

### F125 and F128 are essential for the efficient removal of securin in oocyte meiosis I—where this mechanism is perturbed, oocytes fail to divide

Although the presence of securin is not essential in MI oocytes, its removal at the end of meiosis I is an absolute prerequisite for cell division^30^. We therefore wondered if F125 and F128 function to efficiently remove the non-separase-bound excess of securin ahead of anaphase, mitigating the potential to overwhelm the APC/C in metaphase. Where cyclin B1 is concerned, the majority of oocytes are not capable of removing an excess of cyclin B1 in metaphase if prometaphase destruction is prevented. These oocytes instead arrest in metaphase or early anaphase^44^.

**Fig. 4:**
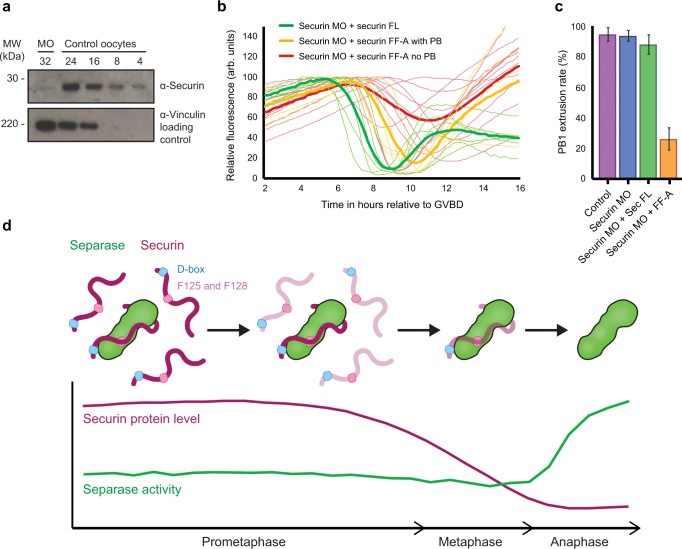
F125 and F128 are essential for the efficient removal of securin in oocyte meiosis I—where this mechanism is perturbed, oocytes fail to divide. **a** Quantification of securin morpholino knockdown. Western blot of control and securin morpholino (MO) injected oocytes collected 5.5 hours post GVBD (numbers of oocytes loaded per lane are indicated). Quantification of protein bands indicates an 87% knockdown of securin in MO-injected oocytes. The lower blot was probed for vinculin and used as a loading control. **b** Securin protein levels were knocked down by morpholino (MO) injection. Oocytes were subsequently injected with either securin FL (green traces, *n* = 6) or securin FF-A (yellow and red traces, *n* = 15). Although securin FL-injected oocytes had normal rates and timings of PB1 extrusion, FF-A-injected oocytes were either delayed (yellow traces) or blocked in MI (red traces). Destruction profiles are relative to GVBD. **c** Quantification of PB1 extrusion rate between control oocytes (purple column, *n* = 50), securin MO-injected oocytes (blue column, *n* = 29), and securin MO-injected oocytes subsequently injected with either securin FL (green column, *n* = 15) or securin FF-A (orange column, *n* = 23). All *n* numbers refer to the number of individual oocytes analysed over a minimum of three independent experiments. Data presented as mean values with error bars ± SEM. **d** A model summarising the mechanism by which a pool of excess non-separase-bound securin is preferentially targeted for destruction in late prometaphase I in oocytes. This mechanism involves both the D-box and a second region within the SIS of securin where F125 and F128 are essential. Following this, a smaller pool of separase-bound securin becomes a destruction target in metaphase.

To test whether the same applies for securin, we knocked down securin protein levels with a morpholino oligo (MO) such that in prometaphase, MO oocytes contained ~13% of the protein level relative to control oocytes (quantified by western blot; Fig. 4a). Despite severe depletion of securin, cell cycle timings and PB1 extrusion rates were normal (Fig. 4b, c). This result agrees with observations made in fixed oocytes, suggesting that cyclin B1-Cdk1 levels are sufficient to compensate for the loss of securin in meiosis I^28^. We then added back an equivalent amount (as quantified by western blot in Supplementary fig. 3a) of either securin FL or securin FF-A to investigate how oocytes progress if the prometaphase destruction mechanism is perturbed. Adding back securin FL showed a destruction profile and PB1 extrusion rate comparable to control oocytes (Fig. 4b, c). In contrast, when securin protein levels were rescued with securin FF-A, destruction was impaired and PB1 extrusion was either delayed by hours or blocked completely. Indeed, only 26% of these oocytes completed meiosis I and extruded a polar body. Whether these oocytes blocked in meiosis I or progressed into meiosis II with a delay in PB1 extrusion was partially dependent on securin FF-A protein levels. In general, those that blocked had higher levels of securin FF-A prior to the onset of destruction. However, as mRNA injection concentrations and expression times were carefully controlled for, we suggest that the higher protein levels seen in some FF-A injected oocytes may suggest a role for F125 and F128 in securin turnover and housekeeping, though this remains a focus of future investigation.

These observations are consistent with a mechanism that allows for the efficient removal of excess securin during prometaphase I in oocytes (Fig. 4d). When this mechanism is perturbed and securin can only be targeted in metaphase, oocytes frequently fail to divide.

## Discussion

Progression through oocyte meiosis is driven by the timely removal of key cell cycle proteins mediated by the APC/C. The segregation of homologous chromosomes in anaphase I requires that both securin and cyclin B1 have been sufficiently depleted^30,55^. Importantly, the degradation of both proteins must be temporally coupled. This ensures that Cdk1 inactivation, triggering meiotic exit, is accompanied by separase activation^30,44^. Active separase then cleaves the cohesin rings distal to centromeres that hold homologous chromosome pairs together during meiosis I^56^. If securin is not degraded in time, the oocyte arrests in metaphase I and cell division fails^30^. Here, we reveal the existence of a novel mechanism of degradation that functions to ensure the removal of excess securin ahead of metaphase.

We demonstrate that in meiosis I, mouse oocytes contain an excess of securin over separase. Furthermore, we show that the oocyte’s destruction machinery is not capable of removing this securin excess in metaphase alone. This necessitates a mechanism of securin degradation that takes place in late prometaphase. In addition to the D-box, two phenylalanine residues (F125 and F128) within the C-terminus of securin are essential for this prometaphase period of degradation. These residues are positioned within the SIS of securin^18,20^ and are predicted to be masked when securin is bound to separase. Indeed, when we use a reporter in which securin is directly tethered to separase, degradation is delayed until metaphase. We therefore suggest that, during late prometaphase, the APC/C primarily targets the non-separase-bound pool of excess securin. Once the oocyte reaches metaphase I, the D-box of securin becomes sufficient for degradation, and a second destruction phase resembling mitotic securin destruction proceeds. Interestingly, while in most eukaryotes the residues corresponding to F125 and F128 in human securin are just downstream of the pseudosubstrate motif (Supplementary fig. 1a), the recently solved *Caenorhabditis elegans* securin-separase structure revealed the corresponding region to be further upstream in the securin sequence and outside the SIS^19^. This suggests that in *C. elegans*, these residues could be involved in securin recruitment to APC/C-Cdc20 regardless of securin-binding state. That this region in *C. elegans* retains high sequence similarity to the equivalent region in the SIS of human securin, despite different positioning within the protein, could hint at the importance of these residues for their alternative function in the regulation of degradation.

We propose that, in oocytes, the majority of non-separase-bound securin is removed in prometaphase, while a smaller pool of separase-bound securin is only targeted in metaphase. By this strategy, the cellular destruction machinery is less likely to become overwhelmed by an excess of substrate during the final stages of chromosome segregation. When we inhibit prometaphase securin destruction by replacing excess endogenous securin with a mutant lacking F125 and F128, the majority of oocytes fail to complete meiosis I.

The mechanism by which free securin is recruited to the APC/C in prometaphase is unclear. Notably, though, initiation of securin destruction in late prometaphase I coincides with the transition from Cdh1- to Cdc20-mediated APC/C activation^55,57^. It has previously been observed that APC/C-Cdh1 activity mediates securin turnover in early prometaphase I^57^. However, it is only after Cdc20 becomes the primary APC/C co-activator at ~6 hours post GVBD that a significant loss of securin protein is observed^30,43^. It is therefore tempting to speculate that the cue for prometaphase destruction of securin and cyclin B1 may be a switch in APC/C co-activator, perhaps mediated by a change in the phosphorylation status of the APC/C. Indeed, two recent studies in mouse oocytes demonstrated that both securin and cyclin B1 are stabilised in oocytes lacking cyclin B3; a B-type cyclin that is highly expressed and essential in the germline but dispensable in mitotic divisions^48,58^. In *Drosophila*, cyclin B3-Cdk1 has been shown to phosphorylate APC3, promoting association between Cdc20 and the APC/C^59^.

Exactly how F125 and F128 in securin interact with the APC/C during prometaphase I is unclear. Interestingly, this region in securin does not share sequence similarity with the PM motif in cyclin B1^44^. Therefore, though their destruction timings are coupled, it seems likely that the mechanisms by which they are targeted are distinct. Indeed, when we inhibit SAC activity in oocytes, while two phases of cyclin B1 destruction are no longer apparent, securin FL is still destroyed in preference to securin lacking these key phenylalanines. These data suggest that, while the two phases of securin destruction have different sensitivities to the SAC, the presence or absence of SAC activity is not the only factor timing these destructions. We suggest that similar to other prometaphase APC/C substrates, F125 and F128 could be involved in direct interaction with APC/C-Cdc20. Notably, cyclin A2 destruction in oocytes initiates in early prometaphase I (Supplementary fig. 5a)^48,60^. This is likely mediated by a combination of its ABBA, D-box, and KEN box motifs as well as direct targeting to the APC/C through its Cdk-associated cofactor Cks^41,42,61^. Interestingly, cyclin A1, which lacks an N-terminal KEN box, is instead targeted 5 hours post GVBD, consistent with Cdh1 being the primary APC/C co-activator in early prometaphase I^60^. It is worth noting that the region of securin containing F125 and F128 bears similarity to the ABBA motif in cyclin A, suggesting that this region of securin may interact with Cdc20 through the same interaction mode as observed in the crystal structure of Acm1(ABBA)-Cdh1^62^ (Supplementary Fig. 5b–e). However, given that immunoprecipitation experiments are not practical using mammalian oocyte lysates, we have been unable to test this so far.

Our study is not the first to report a differential handling of separase-bound and free securin by the APC/C. Hellmuth et al.^54^ previously reported that in HeLa cells, separase-bound securin is dephosphorylated by PP2A and thus stabilised. Free securin on the other hand was found to be in a phosphorylated state, enhancing its ubiquitination by the APC/C. Importantly though, degradation of both separase-bound and free securin in Hela cells takes place in metaphase. In contrast to these findings in mitosis, our data suggest that, in mouse oocytes, phosphorylation could slightly delay the degradation of both free and separase-bound pools of securin. Therefore, while this delay does not account for the major temporal difference between free and separase-bound securin destruction, phosphorylation may still fine-tune the degradation timing of securin in oocytes.

While the removal of an excess of securin is clearly essential for meiotic progression, it is not clear why such excess is present to begin with. When securin protein levels are knocked down in mouse oocytes, meiosis I proceeds with normal timing and without segregation errors^28,29^. This is due to the existence of compensatory mechanisms of separase inhibition in meiosis I. Both securin- and cyclin B1-Cdk1-mediated separase inhibition must be perturbed simultaneously in order to see an increase in segregation defects^28^. In contrast, securin plays an important role both before and after meiosis I in mouse oocytes. In prophase I, securin functions to buffer APC/C-Cdh1-mediated destruction of cyclin B1 during prophase arrest^63^. While in meiosis II, cyclin B1-Cdk1 is no longer able to compensate for depleted securin and severe segregation defects are observed^28,29^. It is therefore plausible that an excess of securin in meiosis I could ensure a threshold level throughout female meiosis in order to support these roles. It should be noted however that neither role is absolutely essential since female securin knockout mice are not sterile, suggesting that in these mice alternative pathways may operate in their place^64^.

An excess of securin may also be beneficial in oocytes from aged mice where cohesin levels are significantly reduced^65–70^. Compounding problems of cohesin loss, oocytes from aged mice destroy securin more rapidly and to a greater extent than oocytes from young mice^71^. As a consequence, separase inhibition is incomplete in these cells, sister chromatid cohesion is lost prematurely, and segregation defects are more frequent. Human oocytes may additionally benefit from an excess of securin in meiosis I, given that separase must be inhibited over a prometaphase period that lasts 14–18 hours, compared with 4.5–6.5 hours in mouse^72–74^.

We conclude that an excess of non-separase-bound securin is present in mouse oocytes and must be removed in prometaphase I. If prometaphase destruction is inhibited, the APC/C is likely overwhelmed by the abundance of the substrate in metaphase, resulting in a stalled meiosis. This period of destruction in prometaphase necessitates a novel mechanism whereby non-separase-bound securin is preferentially targeted by the APC/C. This targeting involves two conserved phenylalanine residues which are likely masked in separase-bound securin. By this strategy, separase-bound securin is preserved, likely allowing for a switch-like activation of separase, essential for the fidelity of anaphase.

## Methods

### Contact for reagent and resource sharing

Further information and requests for resources and reagents should be directed to Suzanne Madgwick (suzanne.madgwick@newcastle.ac.uk).

### Gamete collection and culture

Four-to-10-week-old female, outbred, CD1 mice (Charles River) were used. All animals were handled in accordance with ethics approved by the UK Home Office Animals Scientific Procedures Act 1986. However, given that mice did not undergo a ‘procedure’ as defined by the Act, the project did not require Home Office Licensing. The reason for animal use was instead approved and governed by Newcastle University’s Comparative Biology Centre Ethics Committee; Study Plan Reference Number 699; AWERB Approval Reference Number; 663. GV stage oocytes were collected from ovaries punctured with a sterile needle and stripped of their cumulus cells mechanically using a pipette. For bench handling, microinjections and imaging experiments, oocytes were cultured at 37°C in medium M2 (Sigma) supplemented where necessary with the addition of 30 nM 3-isobutyl-1-methylxanthine (Sigma) to arrest oocytes at prophase I. Data were only collected from oocytes that underwent GVBD with normal timings and had a diameter within 95–105% of the population average. Where destruction profiles are displayed, *n* is the number of oocytes from which the data have been gathered. To ensure reproducibility, oocyte data sets were gathered from a minimum of three independent experiments. For each independent experiment, both control and treatments groups were derived from the same pool of oocytes, collected from a minimum of two animals. Oocytes were selected at random for microinjection. Where necessary, and at the times indicated, nocodazole (Sigma) was added to the media at a final concentration of 150 nM, Mps1 inhibitor reversine (Sigma) at 100 nM^75^, the APC/C inhibitor ProTAME (Boston Biochem) at 1.5 µM, and cycloheximide (Sigma) at 10 μg/ml^76^. For confocal imaging, SiR Hoechst was added to media 30 minutes prior to imaging at a final concentration of 250 nM^77^.

### Preparation of cRNA constructs for microinjection

Wild-type human securin, separase, cyclin B1, Map4, and the separase biosensor (a gift from Jan Van Deursen) were amplified by PCR. Mutations within securin and cyclin B1 were generated by primer overhang extension PCR. A complete list of primers used, including names and sequences, can be found in Supplementary Data 1. Following this, we performed Sequence and Ligation Independent Cloning^78^ using modified pRN3 vectors to generate either a construct with no further tags (the separase biosensor) or constructs coupled to a VFP (all other sequences)^79^. Resultant plasmids were linearised and cRNA for microinjection was prepared using a T3 mMESSAGE mMACHINE kit according to the manufactures instructions (Ambion Inc.). Maximal stability was conferred on all cRNA constructs by the presence of a 5′-globin UTR upstream and both 3’UTR and poly (A)–encoding tracts downstream of the gene. cRNA was dissolved in nuclease-free water to the required micropipette concentration. The cyclin B1 FL construct shown in Fig. 2c contains a Y170A mutation which prevents Cdk1 binding^44^. This reporter is targeted for destruction in time with wild-type cyclin B1 FL but crucially does not affect endogenous Cdk1 activity.

### Knockdown of gene expression using morpholinos

A Morpholino antisense oligo designed to recognise the 5′-UTR of securin (sequence: GATAAGAGTAGCCATTCTGGATTAC; MO; Gene Tools) was used to knock down gene expression. As per the manufacturer’s instructions, the oligo was stored at room temperature, heated for 5 minutes at 65°C prior to use, and loaded at a micropipette concentration of 1 mM.

### Microinjection and imaging

Oocyte microinjection of the MO and construct mRNAs was carried out on the heated stage of an inverted microscope fitted for epifluorescence (Olympus; IX71). In brief, fabricated micropipettes were inserted into cells using the negative capacitance overcompensation facility on an electrophysiological amplifier (World Precision Instruments). This procedure ensures a high rate of survival (>95%). The final volume of injection was estimated by the diameter of displaced ooplasm and was typically between 0.1 and 0.3% of total volume. Reporter construct protein levels were in the range of 25% of endogenous protein levels, as quantified by western blot (Supplementary fig. 3a).

To generate destruction profiles, bright-field and fluorescence images were captured every 10 minutes throughout meiosis I by an inverted Olympus IX71 microscope (fitted for epifluorescence) and CCD camera (Micromax, Sony Interline chip, Princeton Instruments). Regions of interest were manually defined around oocytes and fluorescence levels were measured in 2D. Further analysis was carried out using MetaFluor software (version 7.7.0.0; Molecular Devices). All experiments were performed at 37°C.

Confocal images (including all experiments using the separase biosensor) were captured using a Zeiss LSM-800. Oocytes were imaged at 10 minute intervals through 20 + Z-sections over a 12-hour period from GV stage. All experiments were performed in a temperature-controlled, humidified chamber set at 37°C. Bright-field and fluorescent images were recorded in Zen Blue (Zeiss) and processed in Fiji. By this method, all oocytes extruded polar bodies.

### Molecular structure images and multiple sequence alignments

Molecular structure images were generated using the PyMOL Molecular Graphics System, version 1.3 Schrödinger, LLC. Sequence conservation alignments were made by importing protein sequences from Uniprot and aligning in Jalview, version 15.0. The full sequence alignment conservation annotation shown in Fig. 2d is between securin orthologs from *Homo sapiens*, *Mus musculus*, *Sus scrofa*, *Bos Taurus*, *T. alba*, *Xenopus* *laevis* and *Salmo salar*. Alignment conservation annotation is a quantitative index reflecting the conservation of the physico-chemical properties for each column of the alignment. The tallest yellow columns represent sequence alignment positions with the highest conservation. Shorter columns with increasingly darker shades of brown represent sequence positions with decreasing conservation. All figures were prepared in Adobe Illustrator CC, version 17.1.0.

### Mitotic cell cultures

Standard laboratory HeLa cells were used for immunoprecipitation experiments and cultured in flasks at 37°C, 5% CO_2_ in Dulbecco’s Modified Eagle Medium (DMEM) (Lonza) with 10% fetal bovine serum (FBS, Life Technologies) and antibiotics. Once cell coverage reached ~90%, flasks were treated with 100 nM nocodazole for a 16-hour incubation period. Metaphase cells were then collected by mechanical shake-off and lysed. MEFs were used as western blotting standards and were a gift from Neil Perkins. MEFs were isolated as follows; internal torso connective tissue from 13.5-day embryos was washed in sterile PBS and minced in 1× Trypsin (Invitrogen) for 15 min at 37°C. Following repeated pipetting to break up large tissue fragments, the cell pellet was resuspended in DMEM (Lonza) supplemented with 20% FBS (Gibco, Paisley, UK) and 50 U/ml penicillin/streptomycin (Lonza), and incubated at 37°C in a 5% CO_2_ humidified atmosphere. Once cells reached 90% confluency, they were sub-cultured in 75cm2 flasks and considered as passage 1. Cells were then cultured following the standard 3T3 protocol^80^. Cells were considered immortalised beyond passage 14, but not used in experiments beyond passage 25. Metaphase cells were then collected by mechanical shake-off following an 8-hour incubation period in 100 nM nocodazole.

### Immunoprecipitation

HeLa cells transfected with securin FL, securin FF-A or empty mVenus N1 transfection vector were synchronised and collected as described above. Cells were then lysed in 50 mM Tris-HCl, pH 7.8, 150 mM NaCl, 0.5% NP-40 plus protease inhibitor cocktail (Roche) for 30 min on ice and clarified by a 12,000 × *g* spin for 20 min at 4°C. Complexes were immunoprecipitated for 90 minutes at 4°C with GFP-Trap beads (Chromotek). After five washes in lysis buffer, proteins were eluted from beads by incubating for 10 minutes at 95 °C in sample buffer. The supernatant was then analysed by immunoblotting.

### Immunoblotting

Mitotic MEF cell lysates were prepared using Laemmli buffer following mechanical shake-off of metaphase cells. Oocytes were collected 5.5 hours after GVBD ± 15 min and lysed in Laemmli buffer. Sodium dodecyl sulphate–polyacrylamide gel electrophoresis and immunoblotting were carried out by standard procedures. Immunoblot membrane sections were incubated for 16 hours at 4°C with either anti-securin (Abcam, AB3305; 1:5000), anti-separase (Abnova, 6H6; 1:5000), or anti-vinculin (Cell Signaling, E1E9V; 1:2000). Non-fat milk (5%) was used as a blocking solution and anti-mouse IgG (7076P2; Cell Signaling; 1:20000 with AB3305 and 1:50000 with 6H6) and ECL Select (RPN2235; GE Healthcare) were used as secondary detection reagents. ECL Select detection reagents were specifically used to produce quantifiable chemiluminescent signals with a broad linear dynamic range. Membranes were exposed to Hyperfilm x-ray film (Amersham Biosciences) and developed using a SRX101 film processor (Konica). Exposure time depended on the strength of the signal. Immunoblots are representative of at least three independent blots. Uncropped versions of all blots shown in figures can be found in the source data file.

### Quantification and statistical analysis

Real-time destruction profiles were recorded in MetaFluor (Molecular Devices) and data were automatically logged in Excel. By taking an average VFP intensity reading from a defined region of interest around the oocyte, fluorescence intensity was plotted over time and oocyte data sets were aligned at PB1 extrusion unless otherwise stated. Average polar body extrusion timings were identical between experimental groups unless otherwise stated. Fluorescence data values are arbitrary. In order to give the destruction profile of each oocyte equal weighting, when generating a mean treatment destruction profile, all individual data sets were normalised prior to calculation (taking the maximum point of fluorescence prior to protein destruction as 100 arbitrary units). However, like the examples shown in Figure S1i, we also compared all raw traces, confirming that in each case experiment, the order and pattern of construct destruction remained the same, regardless of the data handling method. All comparisons only include data from oocytes expressing constructs to similar levels (see supplementary fig. S1i for example). Given the 1:1 ratio of the securin to VFP component of each construct, we reasoned that comparable fluorescence levels equate to comparable molar amounts. Oocytes with either excessive starting amounts of reporter proteins, or with excessive translation rates were discounted. All mean destruction traces have associated data sets in individual oocytes.

Mean cleavage profiles for separase biosensor experiments were produced in Fiji by creating a clipping mask to the DNA using the far red signal emitted by SiR-DNA treatment. The eGFP and mCherry intensity readings from the clipping mask were then plotted over time and aligned at PB1 extrusion. eGFP/mCherry ratios were calculated in Excel.

When only two constructs are compared, we have displayed both the mean trace and individual traces. Where three or more constructs are compared, for clarity, only the mean trace is shown with SEM error bars.

### Structural modelling of the securin_FF_-Cdc20 complex

The crystal structure of human Cdc20 (PDB accession 4GGC [10.2210/pdb4GGC/pdb]^81^) was superposed onto yeast Cdh1 of the Acm1-Cdh1 crystal structure (PDB accession 4BH6 [10.2210/pdb4BH6/pdb]^62^) with an rms deviation of 0.60, using Phenix superpose^82^. The human securin sequence corresponding to amino acids 124–142 was threaded onto the Acm1 chain, mapping to amino acids 60–79, with one amino-acid gap between residues 65 and 67 (as indicated in supplementary fig. 5), using Coot^83^. Protein-peptide docking, with optimisation of the peptide backbone and rigid body orientation, was performed using the Rosetta FlexPepDock web server^84,85^. Models were ranked according to their Rosetta full-atom energy score; the top ten models showed scores of between −593.039 and −590.110, with rms deviations from the initial model of between 1.911 and 3.122. The top ten models were superposed and displayed together, and the top-ranked model was displayed superposed with the Acm1-Cdh1 crystal structure (PDB accession 4BH6 [10.2210/pdb4BH6/pdb]^62^), using the PyMOL Molecular Graphics System, Version 2.0 Schrödinger, LLC.

### Reporting summary

Further information on research design is available in the Nature Research Reporting Summary linked to this article.

## Supplementary information

Supplementary Figures 1–5

Supplementary Data 1. List of primer names and sequences used.

Reporting Summary

## Data Availability

All data generated or analysed during this study are included in this published article (and its supplementary information files). Source data are provided with this paper.

## Source data

Source Data
