# Supplementary material for: A prometaphase mechanism of securin destruction is essential for meiotic progression in mouse oocytes
Source: Nat Commun. 2021 Jul 14;12:4322. doi: 10.1038/s41467-021-24554-2 (PMC8280194; doi:10.1038/s41467-021-24554-2)
Supplement: Supplementary file 1 — Supplementary Figures 1–5 [file 41467_2021_24554_MOESM1_ESM.pdf]

Supplementary figure 1

a

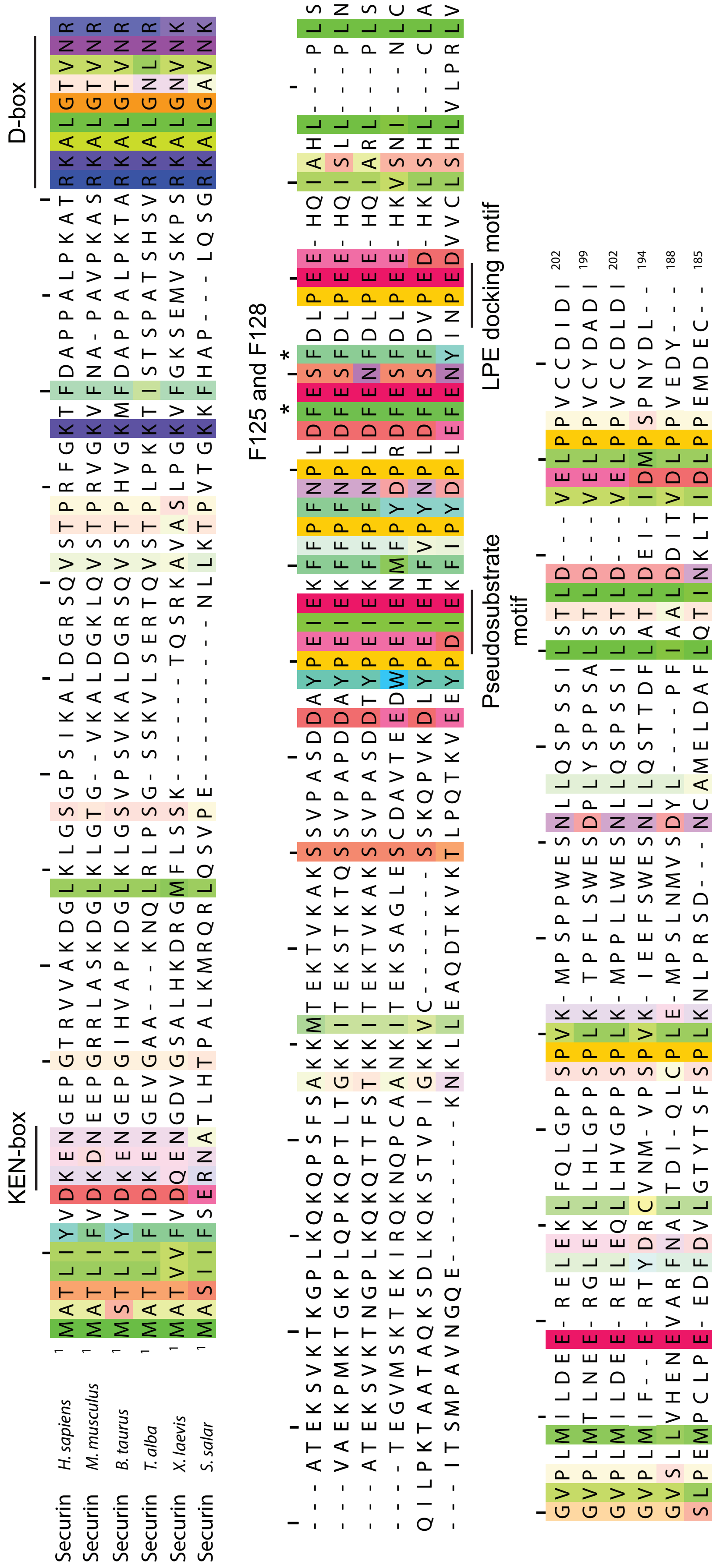

Supplementary figure 1 (continued)

b

Securin FL	109 D A Y P E I E K F F P F N P L D F E S F D L P E E 133
Securin DAYPEIE-A	109 A A A A A A A K F F P F N P L D F E S F D L P E E 133
Securin FFPFNP-A	109 D A Y P E I E K A A A A A A L D F E S F D L P E E 133
Securin DFESFD-A	109 D A Y P E I E K F F P F N P L A A A A A A L P E E 133
Securin FF-A	109 D A Y P E I E K F F P F N P L D A E S A D L P E E 133

c

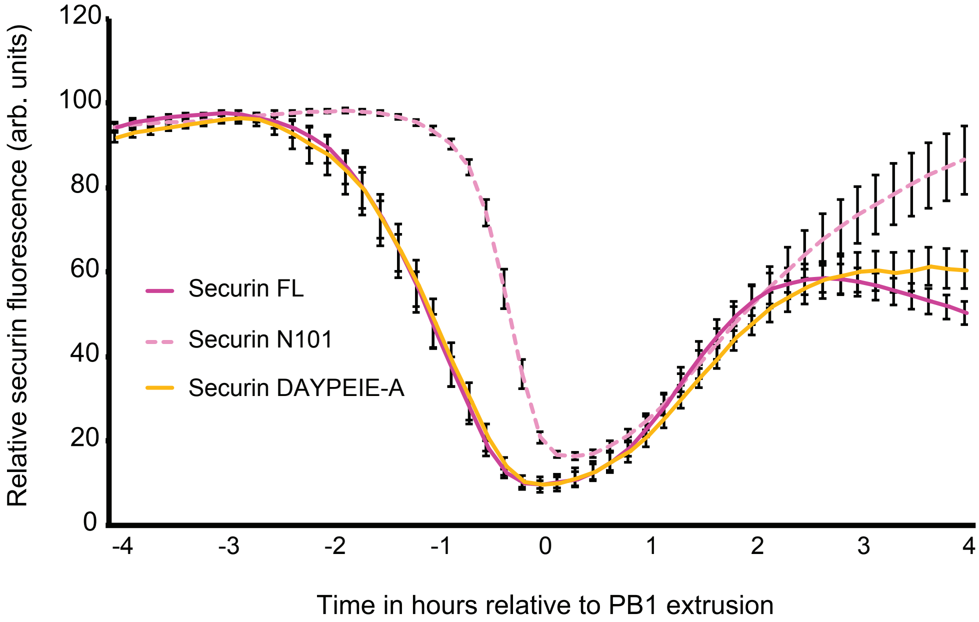

d

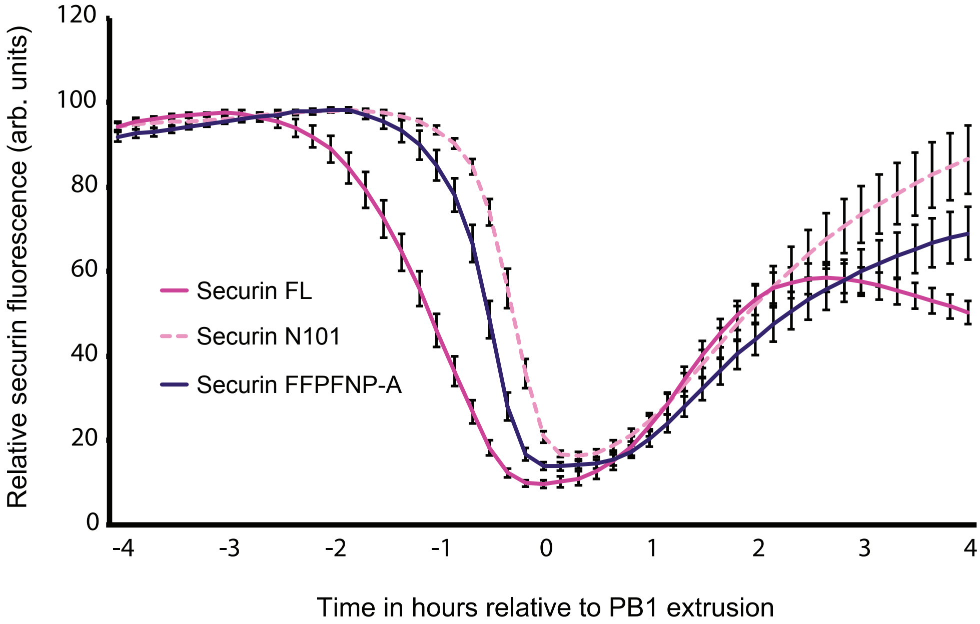

Supplementary figure 1 (continued)

e

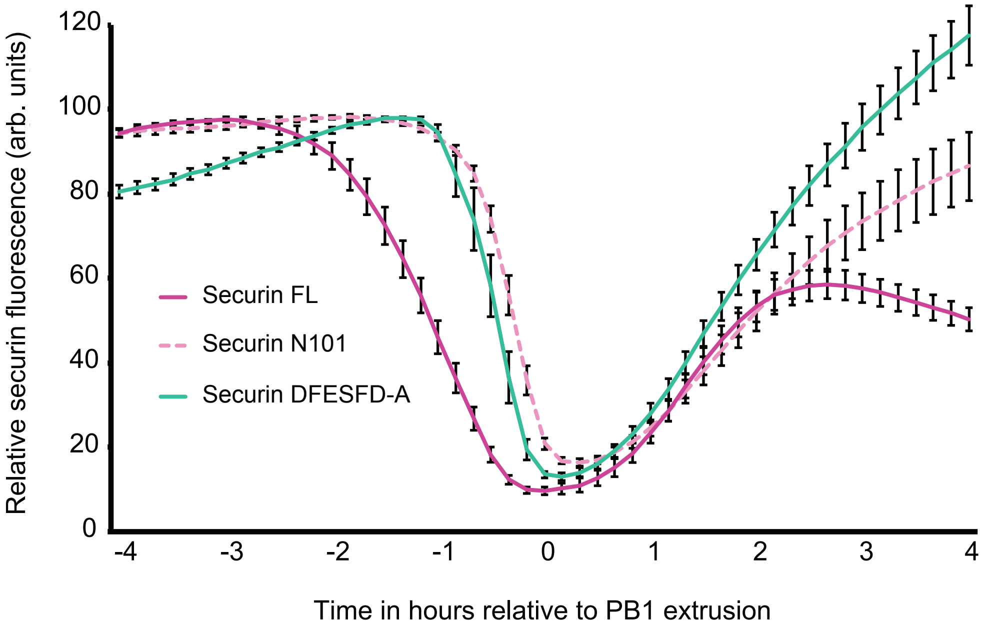

f

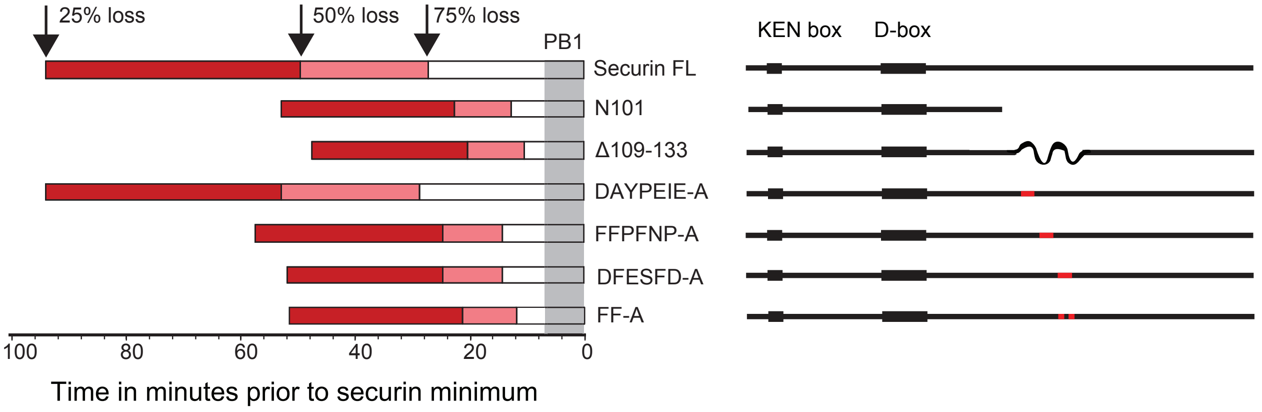

g

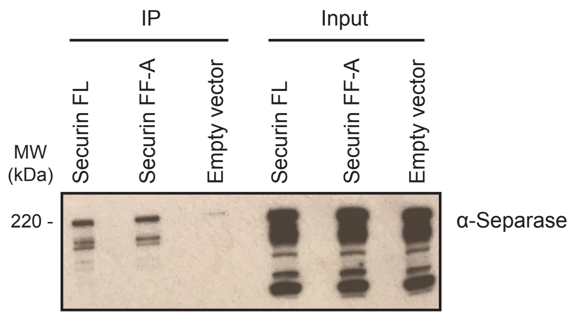

Supplementary figure 1 (continued)

h

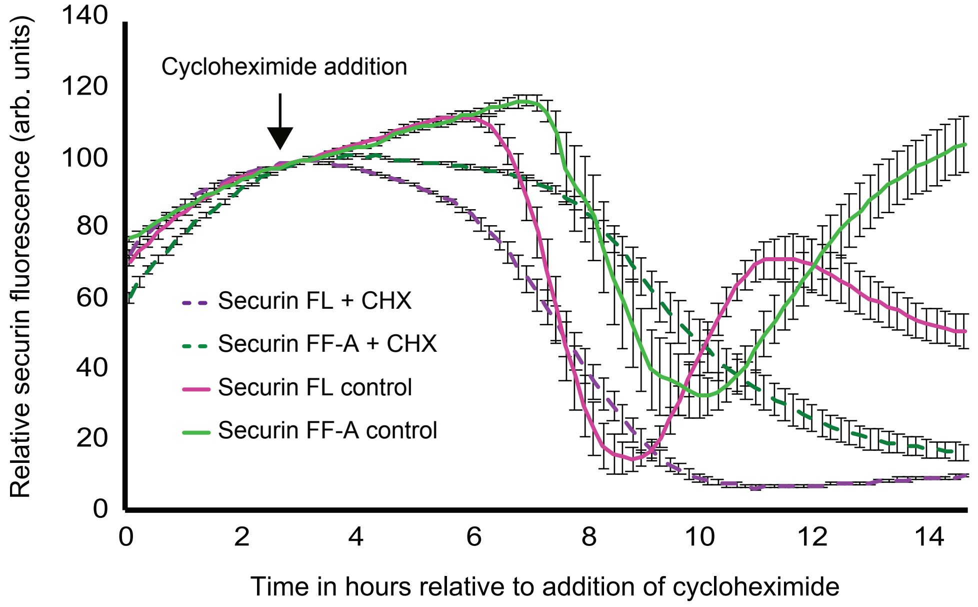

i

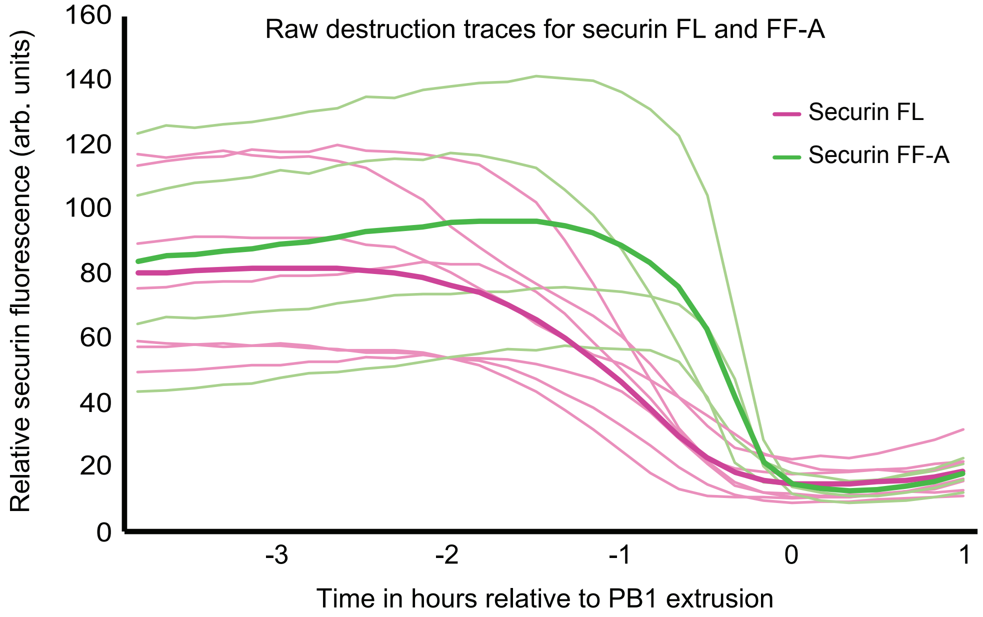

Supplementary figure 1. Destruction profiles of securin mutation constructs leading to identification of the F125 and F128 as essential for prometaphase securin destruction. (a) Full sequence alignment of securin orthologs. Securin residues F125 and F128 are marked with asterisks. (b) Securin residues 109-133 showing sequence detail relative to the nomenclature of all venus-tagged securin mutations assayed in parts c-e. (c) Mean VFP-tagged securin FL (magenta trace, n=25), securin N101 (dashed pink trace, n=23) and securin DAYPEIE-A (yellow trace, n=19) destruction traces. (d) Mean VFP-tagged securin FL (magenta trace, n=25), securin N101 (dashed pick trace, n=23) and securin FFPFNP-A (purple trace, n=23) destruction traces. (e) Mean VFP-tagged securin FL (magenta trace, n=25), securin N101 (dashed pick trace, n=23) and securin DFESFD-A (green trace, n=20) destruction traces. All traces are aligned at PB1 extrusion and error bars = ±SEM throughout. (f) Direct comparison of destruction timings all securin constructs. Schematic representations of constructs are shown down the right. To the left, the length of each bar indicates the destruction timing relative to complete destruction. The open, white bars indicate the point at which 75% of the destruction has taken place. The light red extension to this bar indicates the point at which 50% of the destruction has taken place, followed by a dark red extension indicating the point at which 25% of the destruction has taken place. The period over which PB1 extrusions occur is in grey. (g) Immunoprecipitation of mitotic lysates from HeLa cells transfected with securin FL, securin FF-A, or empty mVenus N1 transfection vector probed for separase. Immunoblot is representative of 2 independent repeats. (h) VFP-tagged securin FL (purple dashed trace, n=24) and securin FF-A (dark green dashed trace, n=27) destruction profiles on addition of 10 μg/ml cycloheximide to inhibit protein synthesis. Mean VFP-tagged securin FL (magenta trace, n=16) and securin FF-A (green trace, n=16) destruction profiles in control oocytes are included as a reference. Traces are aligned to GVBD. (i) Example of raw destruction profiles for VFP-tagged securin FL (magenta) and securin FF-A (green) aligned at PB1 extrusion. Fine traces represent individual oocyte fluorescence levels and heavy traces represent their mean throughout. All n numbers refer to the number of individual oocytes analysed over a minimum of 3 independent experiments.

Supplementary figure 2

a

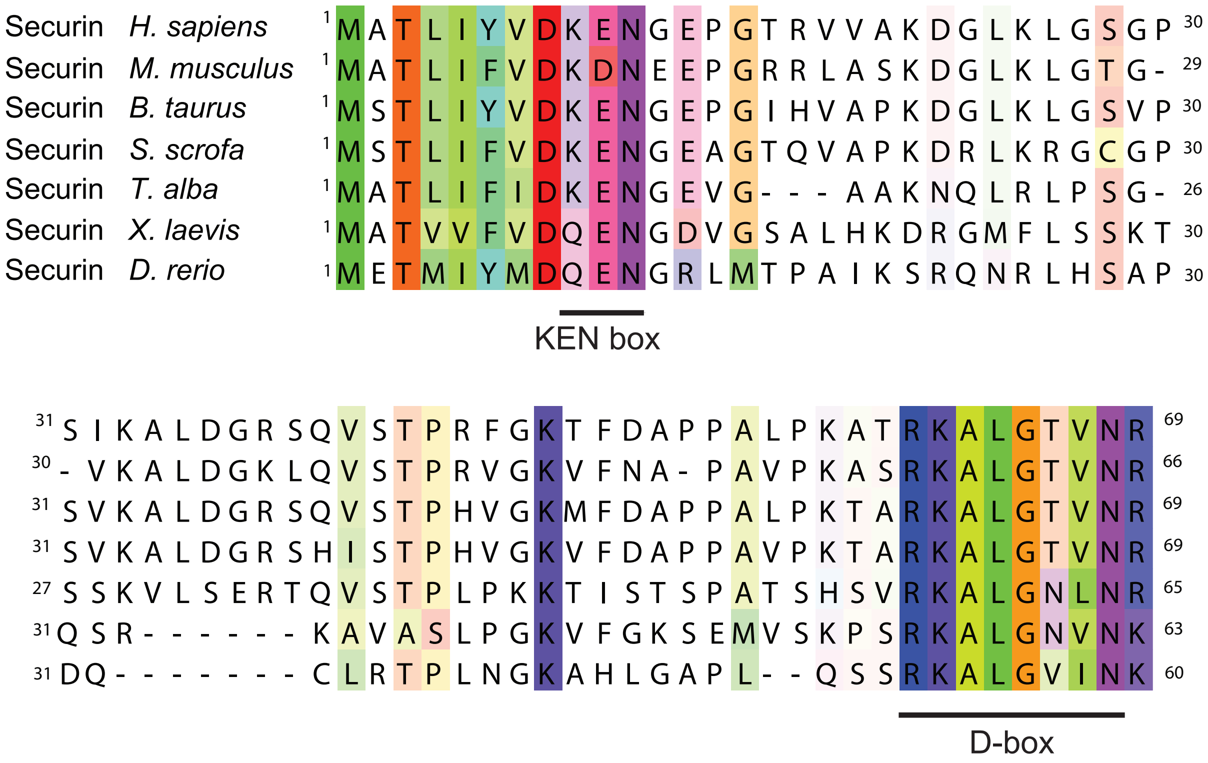

b

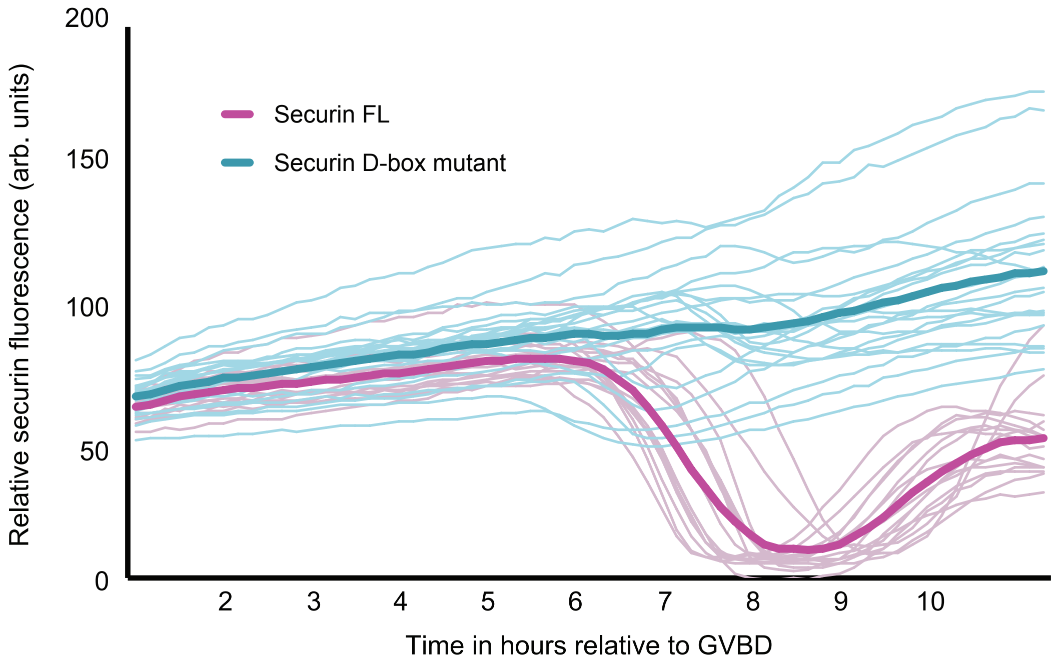

Supplementary figure 2 (continued)

c

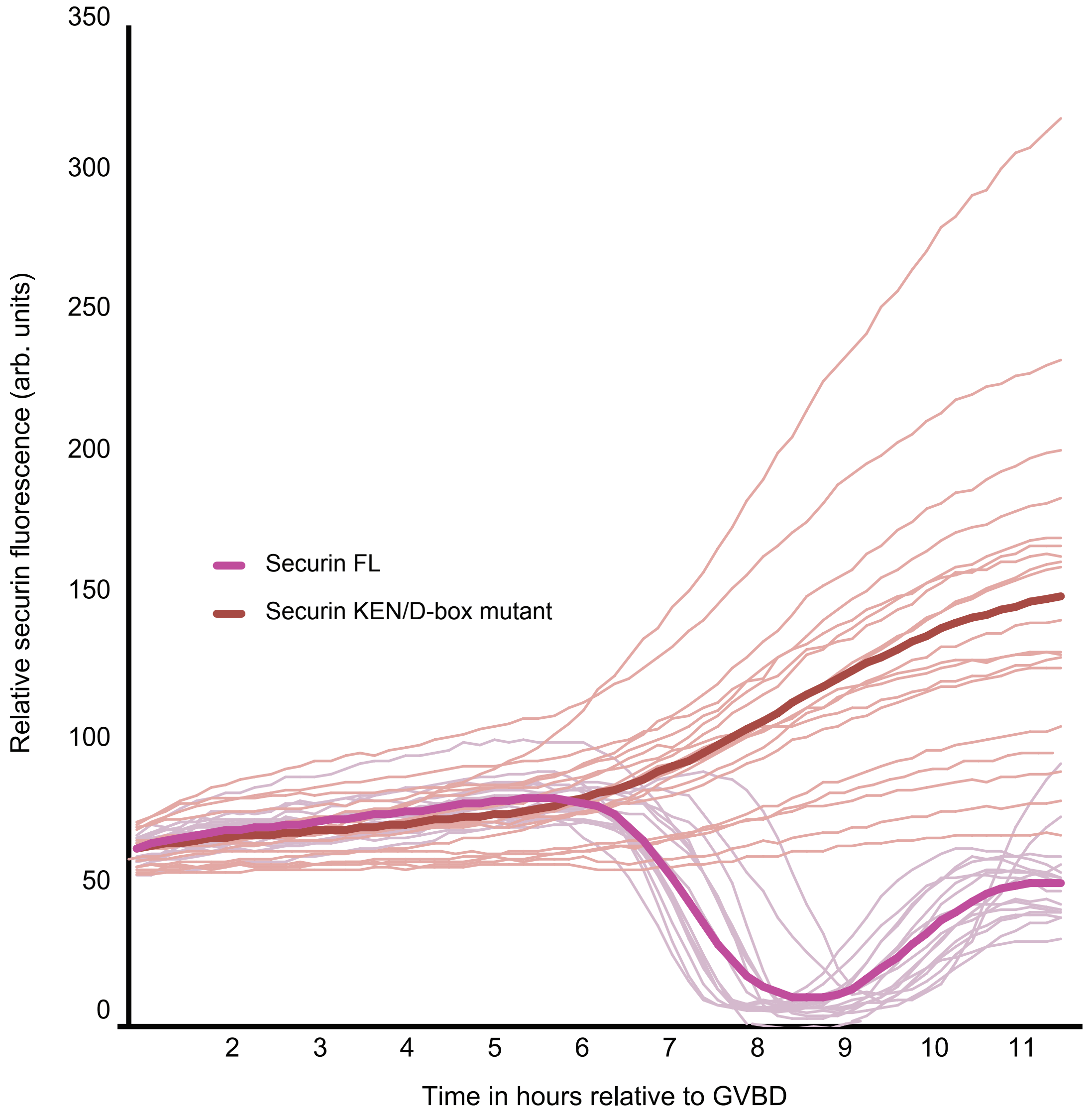

d

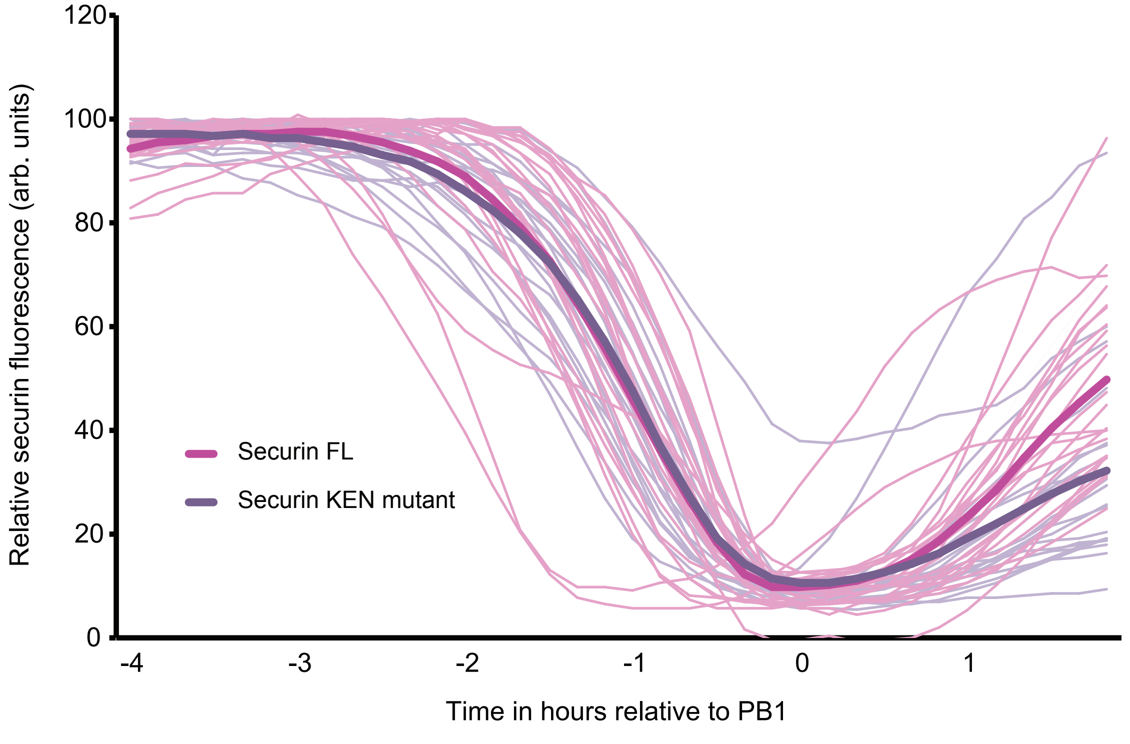

Supplementary figure 2. Meiotic securin destruction is D-box dependent but not KEN box dependent. (a) Alignment of residues 1-69 in securin orthologs containing both KEN box and D-box motifs as indicated. (b) VFP-tagged securin FL (magenta traces, n=16) and securin D-box mutant (light blue traces, n=22) destruction profiles aligned at GVBD (oocytes expressing D-box mutant securin arrest their cell cycle in metaphase do not extrude a polar body). (c) VFP-tagged securin FL (magenta traces, n=16) and securin KEN/D-box mutant (red traces, n=19) destruction profiles aligned at GVBD. (d) VFP-tagged securin FL (magenta traces, n=25) and securin KEN mutant (purple traces, n=21) destruction profiles aligned at PB1 extrusion. Fine traces represent individual oocyte fluorescence levels and heavy traces represent their mean throughout.

Supplementary figure 3

a

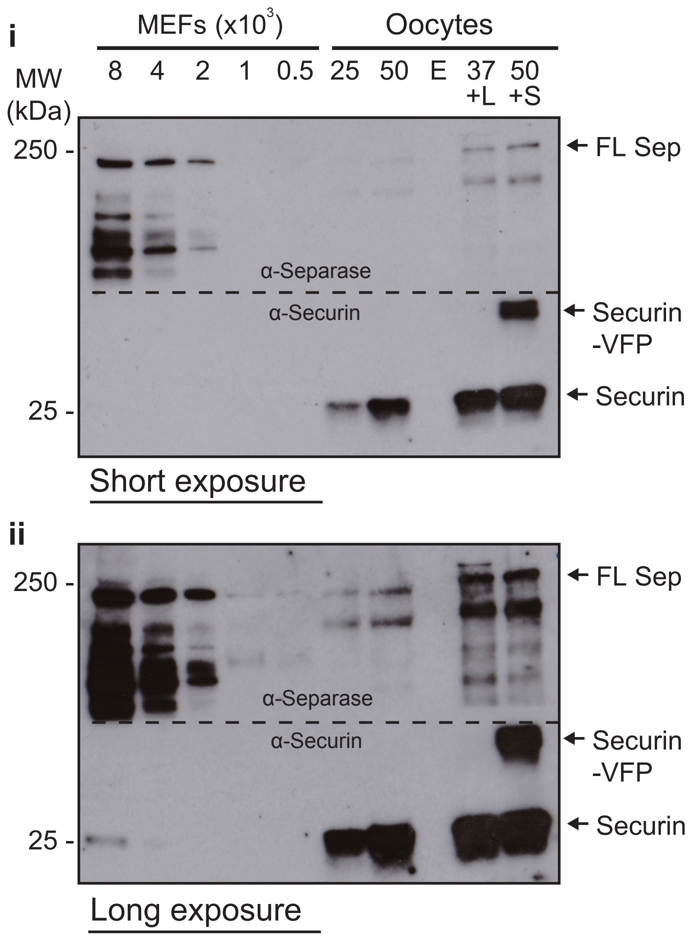

b

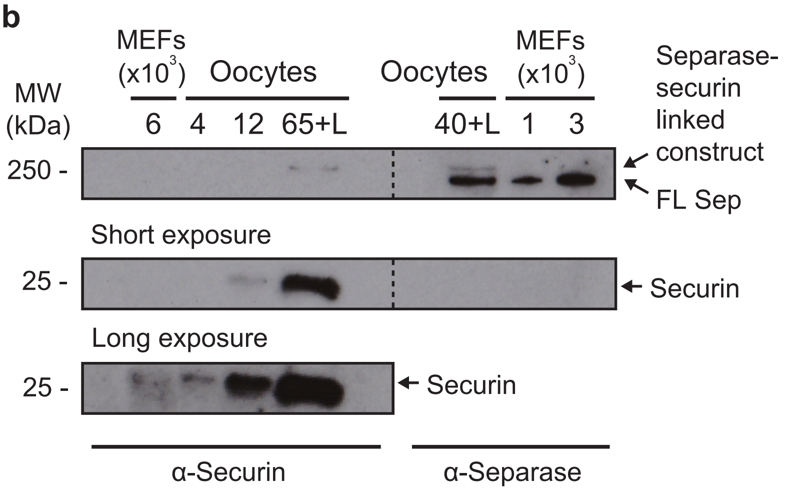

c

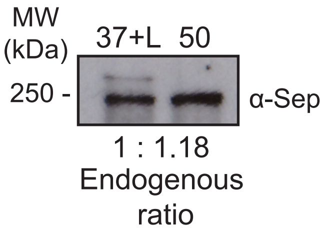

d

Calculation of securin:separase ratio in oocytes

Quantification is from blot in part B

Linked construct securin band intensity in 65 oocytes = 1173
Linked construct separase band intensity in 40 oocytes = 1627
Linked construct separase band intensity in 65 oocytes (1627*(65/40)) = 2644

Therefore in linked construct band with known 1:1 securin separase ratio, separase antibody staining is (2644/1173) = 2.25x more intense

Endogenous securin band intensity in 65 oocytes = 9488
Endogenous separase band intensity in 40 oocytes = 3679
Endogenous separase band intensity in 65 oocytes (3679*(65/40)) = 5978

Endogenous securin band intensity in 65 oocytes corrected for difference between antibody intensities (as detailed above) = 9488*2.25 = 21,385

Estimated securin:separase ratio in oocytes = 21,385/5978 = 3.6 : 1

e

Calculation of securin:separase ratio in MEFs

Quantification is from blot in part B and the calculated securin:separase ratio in oocytes from part D

Endogenous separase in 40 oocytes is equal to the amount of endogenous separase in 1365 MEFs as calculated by MEFs standard curve

Separase in 1 oocyte = 34 MEFs

Endogenous securin in 6,000 MEFs is equal to the amount of endogenous securin in 3.5 oocytes as calculated by oocyte standard curve

Securin in 1 oocyte = 1,739 MEFs

1,739/3.6 (securin:separase ratio in oocytes) = 483
483/34 (separase equivalency between oocytes and MEFs) = 14.2

Estimated securin:separase ratio in MEFS = 1 : 14.2

Supplementary figure 3. Relative abundance of securin and separase protein in mouse oocyte in late prometaphase I and in MEF cells arrested in mitosis. Various western blots were performed using lysates prepared from either MEF cells enriched in mitosis (collected by shake off following incubation in nocodazole), or mouse oocytes collected 5 hours post GVBD (as securin levels are expected to peak in most oocytes). Representative blots are shown from at least 3 independent replicates. Lysates were loaded as indicated. In some cases oocytes were microinjected with mRNA for a linked separase-securin VFP construct (see also fig. 4c); '+L', or mRNA to wild-type mouse securin-VFP at 4x typical injection concentration '+S'. 'E' indicates an empty lane. Membranes were cut as indicated with a black dashed line and incubated with either anti-securin or anti-separase. Note from images (ai and aii), that while separase was readily detectable in MEF cells, we could only ever detect securin in the maximum numbers we were able to load in a single lane. This indicated that securin:separase ratio is vastly different between oocyte meiosis I and early embryonic mitosis (MEF cells). In order to quantify these ratios we made further comparisons using a linked separase-securin construct in which mouse securin and mouse separase were present at a 1:1 ratio (b). Importantly, expression of this tethered construct did not perturb endogenous securin (ai) or separase (c) levels. Our analysis detailed in (d) resulted in an estimated securin:separase ratio in mouse oocytes of 3.6 : 1. We then quantified the ratio in MEF cells as detailed in (e), which resulted in an estimated securin:separase ratio of 1 : 14.2.

Supplementary figure 4

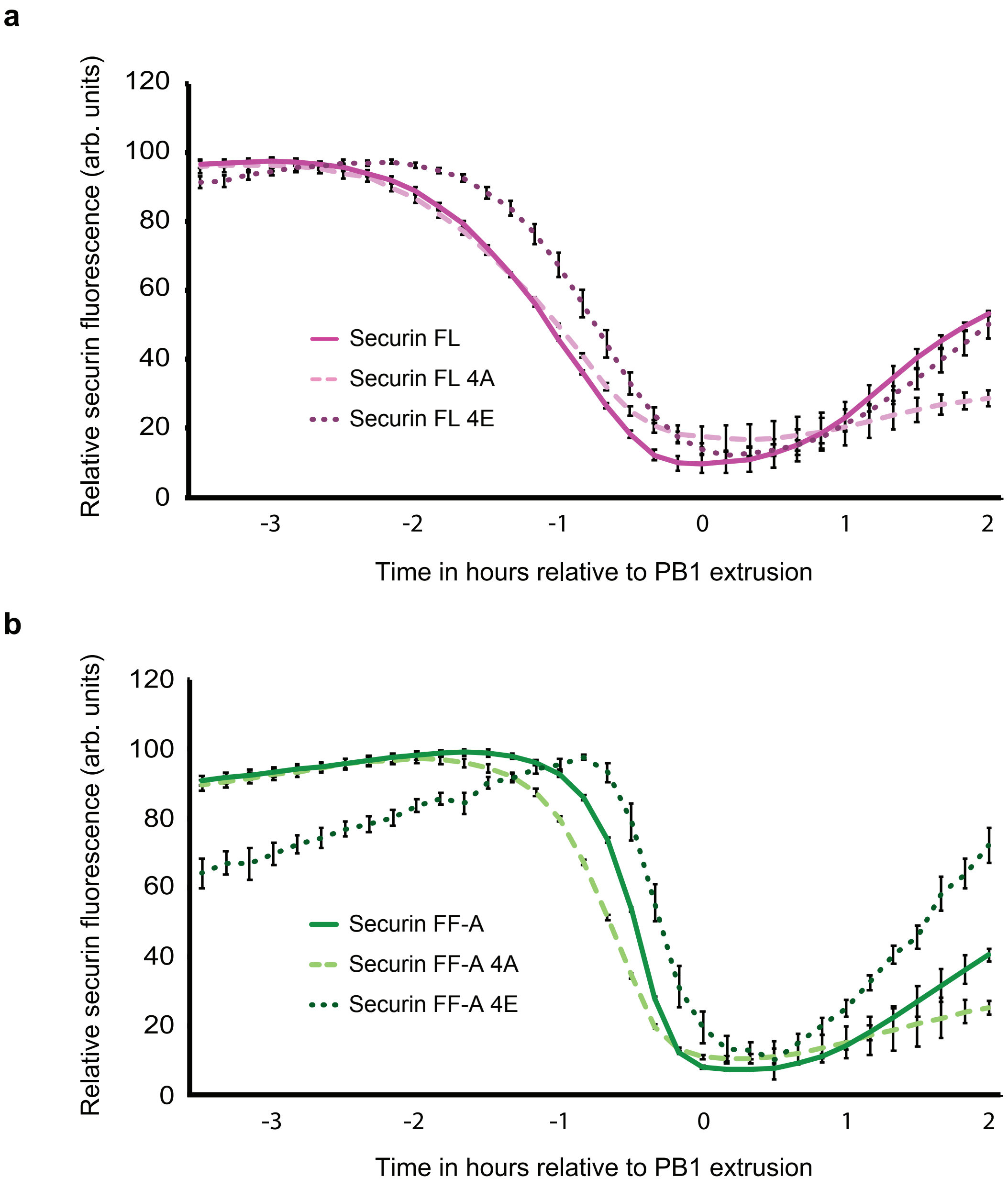

Supplementary figure 4. Phosphomimetic mutations delay securin destruction in oocytes. Phospho-null and phosphomimetic securin FL and securin FF-A were generated by mutating the four key phosphorylation sites in securin identified by Hellmuth et al. (S31, T66, S87 and S89) to either alanine (4A; phospho-null), or glutamic acid (4E; phosphomimetic). (a) Mean VFP-tagged securin FL (magenta trace, n=25), securin FL 4A (pink dashed trace, n = 17) and securin FL 4E (purple dotted trace, n=27). (b) Mean VFP-tagged securin FF-A (green trace, n=20), securin FF-A 4A (light green dashed trace, n=20) and securin FF-A 4E (dark green dotted trace, n=14). Destruction profiles are aligned at PB1 extrusion. All n numbers refer to the number of individual oocytes analysed over a minimum of 3 independent experiments. Error bars = +/- SEM.

Supplementary figure 5

a

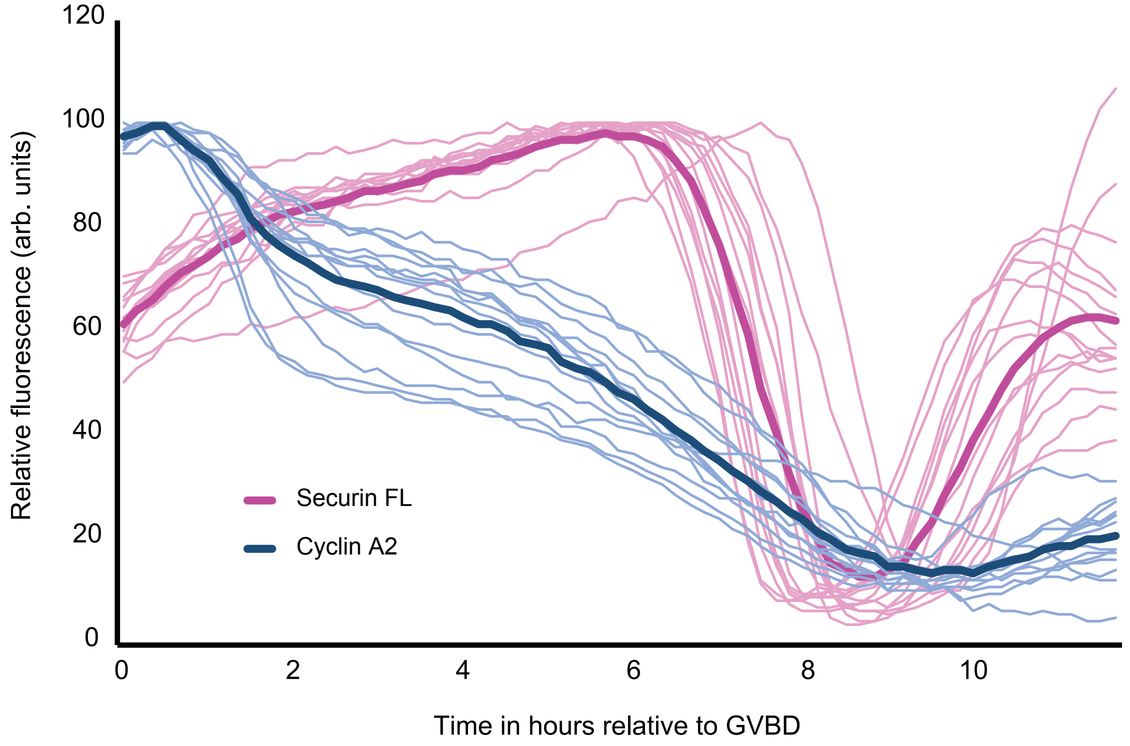

Supplementary figure 5 (continued)

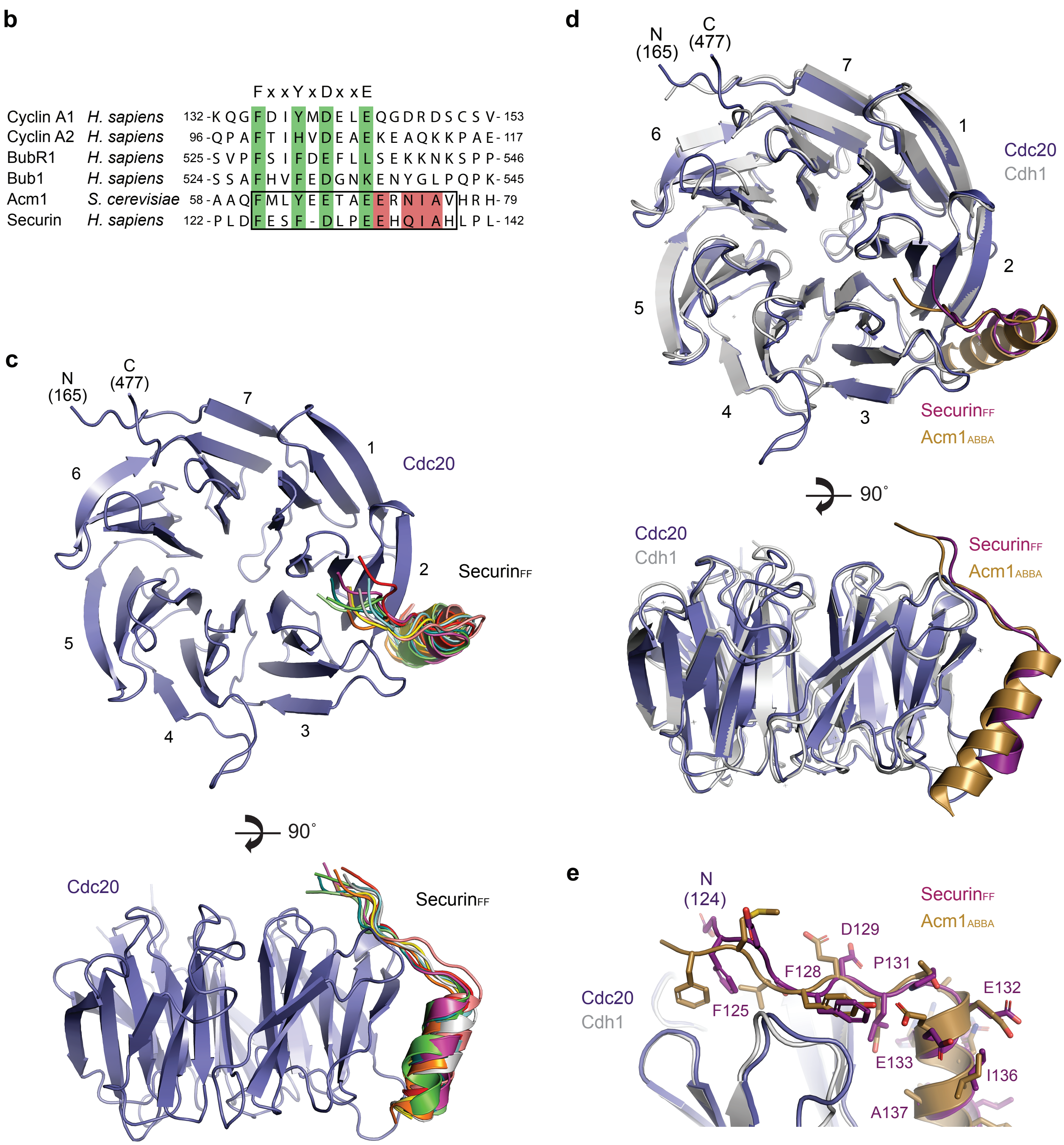

Supplementary figure 5. The region surrounding human securin residues F125 and F128 bears similarity to an ABBA motif. (a) VFP-tagged securin FL (magenta traces, n = 15) and cyclin A2 (blue traces, n = 15) destruction profiles aligned at GVBD. Fine traces represent individual oocyte fluorescence levels and heavy traces represent their mean throughout. (b) Multiple-sequence alignment of ABBA motifs in human Cyclin A1, Cyclin A2, BubR1 and Bub1, and yeast Acm1 with the residues surrounding F125 and F128 in human securin (amino acid boundaries are indicated). The ABBA consensus sequence FxxYxDxxE is shown, with sequences highlighted in green. Additional sequence conservation between Acm1 and securin is shown in red. (c-e) Structural modelling of the securin$_{FF}$-Cdc20 complex using the Acm1$_{ABBA}$-Cdh1 and Cdc20 crystal structures (PDB accessions 4BH6 [http://doi.org/10.2210/pdb4BH6/pdb] and 4GGC [http://doi.org/10.2210/pdb4GGC/pdb]) as templates for protein-peptide docking using Rosetta FlexPepDock. (c) Superposition of the top ten ranked models of securin$_{FF}$-Cdc20 in which Cdc20 is shown in blue and securin$_{FF}$ peptides are shown in a spectrum of colours. (d-e) The top-ranked model of securin$_{FF}$-Cdc20 superposed with the Acm1$_{ABBA}$-Cdh1 crystal structure (PDB accession 4BH6 [http://doi.org/10.2210/pdb4BH6/pdb]), with chains shown in blue (Cdc20), grey (Cdh1), magenta (securin$_{FF}$) and wheat (Acm1$_{ABBA}$). (e) Structural details of the side-chain conformations of securin$_{FF}$ and Acm1$_{ABBA}$, with securin residue numbers indicated.
