# Supplementary material for: A prometaphase mechanism of securin destruction is essential for meiotic progression in mouse oocytes
Source: Nat Commun. 2021 Jul 14;12:4322. doi: 10.1038/s41467-021-24554-2 (PMC8280194; doi:10.1038/s41467-021-24554-2)

Figure 4A - uncropped blots

Anti-securin (Abcam ab3305)

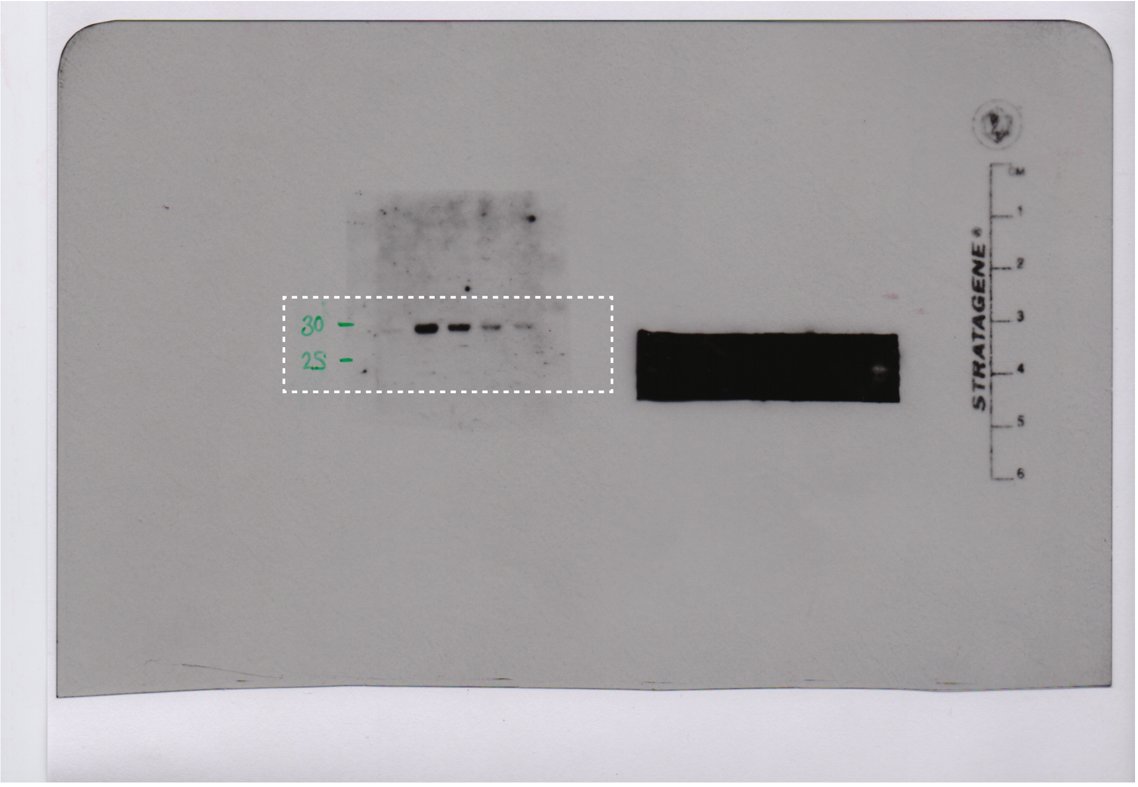

Anti-vinculin (Cell Signaling, E1E9V)

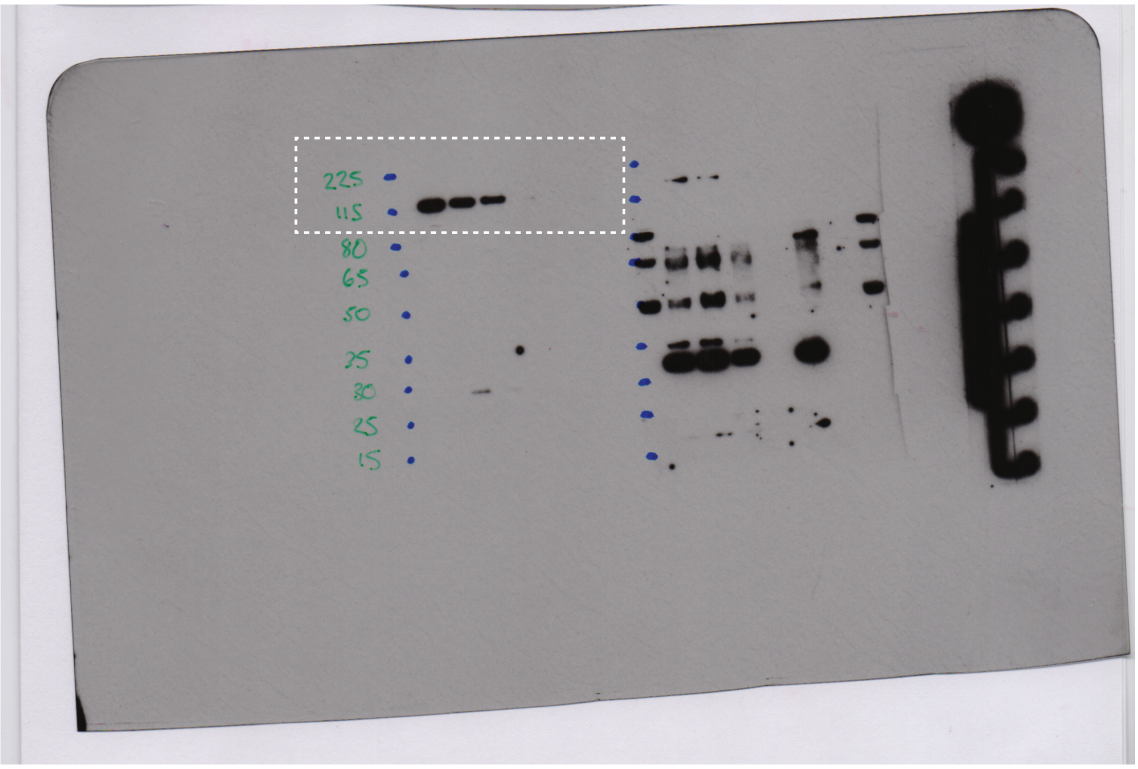

Supplementary figure 1G - uncropped blot

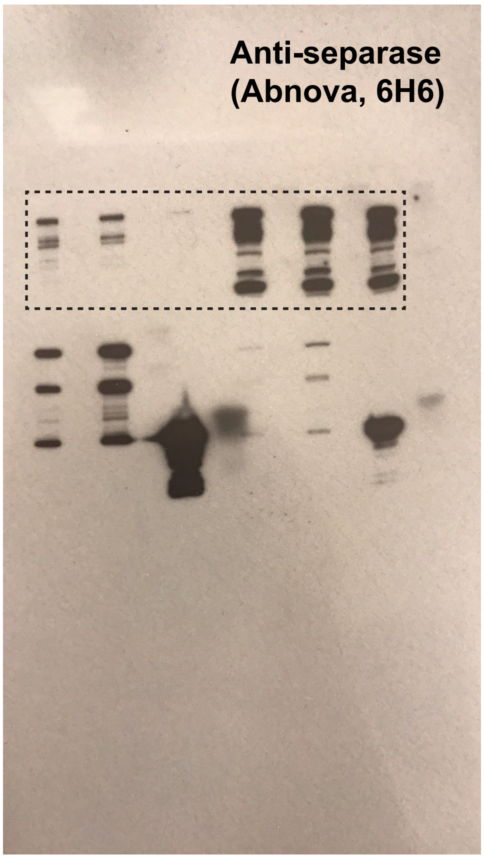

Supplementary figure 3 - uncropped blots

Ai.

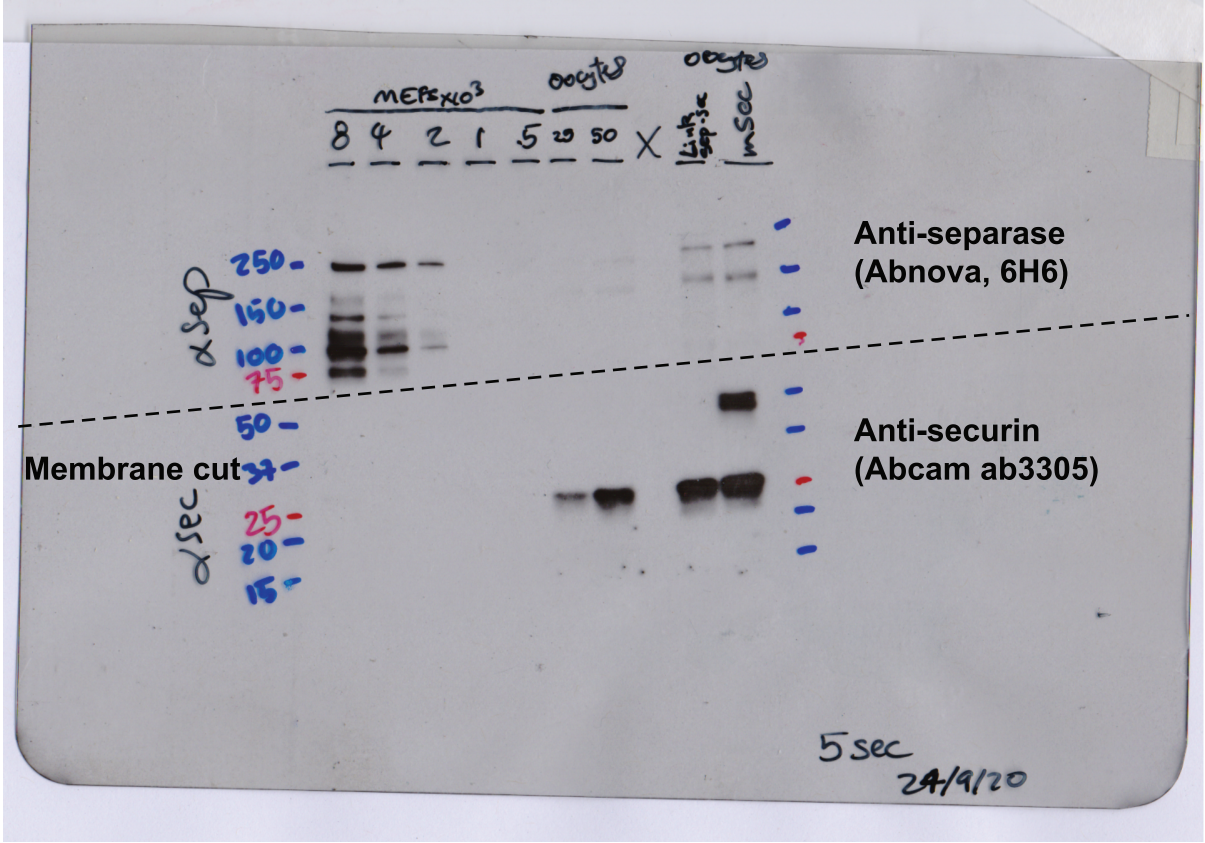

Aii.

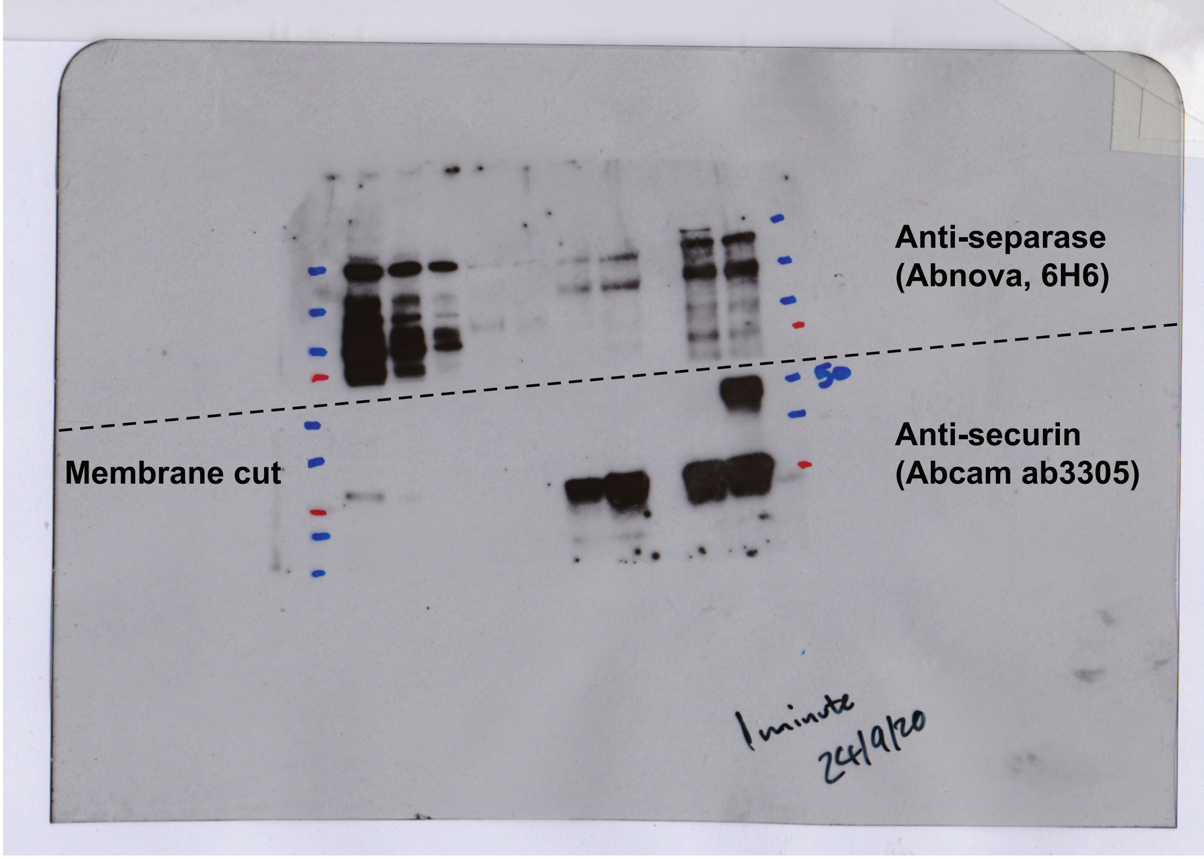

Supplementary figure 3 - uncropped blots (continued)

B.

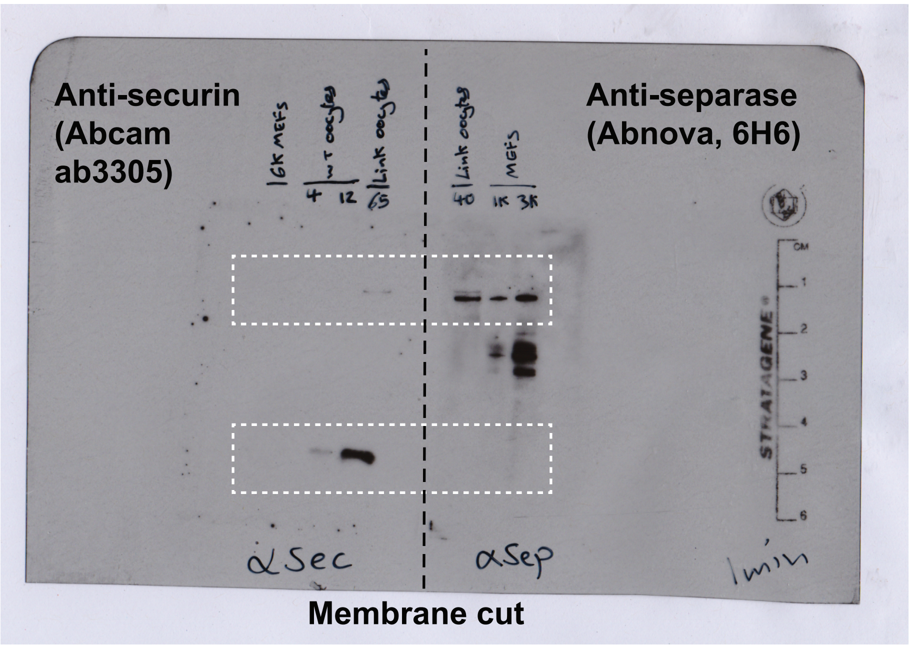

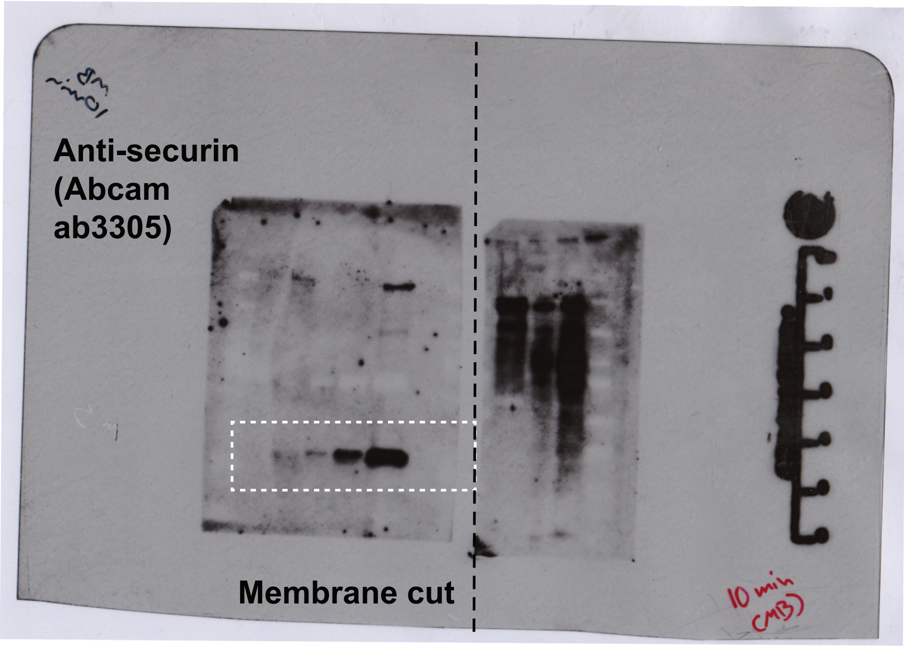

C.

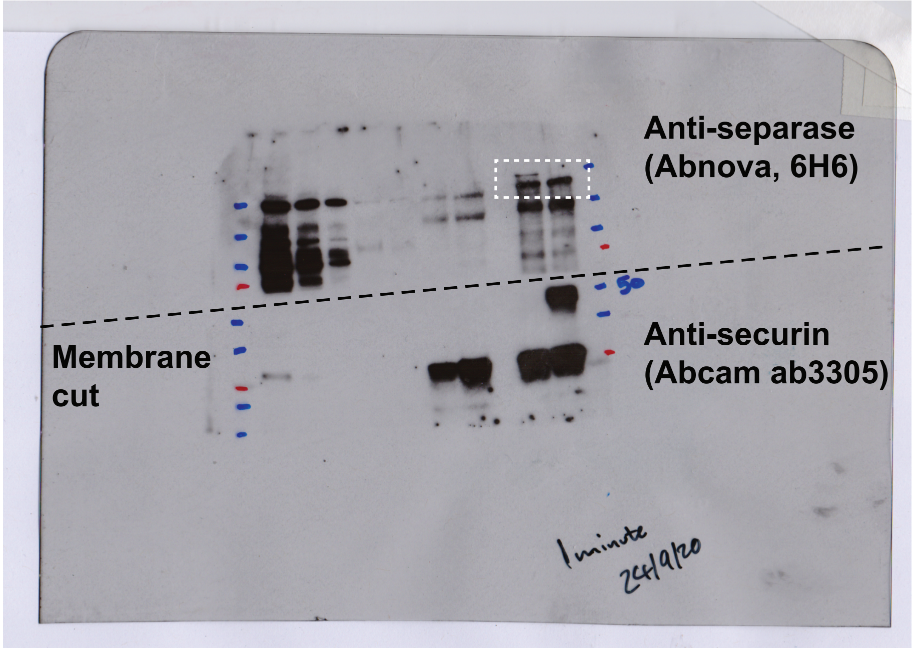

Supplement: Supplementary file 4 — Source Data [file 41467_2021_24554_MOESM4_ESM.zip › Source Data/Source data - uncropped blots.pdf]
